# Recent Advances in Superhydrophobic Materials Development for Maritime Applications

**DOI:** 10.1002/advs.202308152

**Published:** 2024-02-25

**Authors:** Zhao Qing Tang, Tongfei Tian, Paul J. Molino, Alex Skvortsov, Dong Ruan, Jie Ding, Yali Li

**Affiliations:** ^1^ Centre for Smart Infrastructure and Digital Construction School of Engineering Swinburne University of Technology Hawthorn VIC 3122 Australia; ^2^ School of Science, Technology and Engineering University of the Sunshine Coast Sippy Downs QLD 4556 Australia; ^3^ Platforms Division Defence Science and Technology 506 Lorimer Street Fishermans Bend VIC 3207 Australia; ^4^ Department of Mechanical Engineering and Product Design Engineering Swinburne University of Technology Hawthorn Melbourne VIC 3122 Australia

**Keywords:** air‐layer, anti‐corrosion, bio‐inspired, biomimetic, robustness, superhydrophobicity, submersible

## Abstract

Underwater superhydrophobic surfaces stand as a promising frontier in materials science, holding immense potential for applications in underwater infrastructure, vehicles, pipelines, robots, and sensors. Despite this potential, widespread commercial adoption of these surfaces faces limitations, primarily rooted in challenges related to material durability and the stability of the air plastron during prolonged submersion. Factors such as pressure, flow, and temperature further complicate the operational viability of underwater superhydrophobic technology. This comprehensive review navigates the evolving landscape of underwater superhydrophobic technology, providing a deep dive into the introduction, advancements, and innovations in design, fabrication, and testing techniques. Recent breakthroughs in nanotechnology, magnetic‐responsive coatings, additive manufacturing, and machine learning are highlighted, showcasing the diverse avenues of progress. Notable research endeavors concentrate on enhancing the longevity of plastrons, the fundamental element governing superhydrophobic behavior. The review explores the multifaceted applications of superhydrophobic coatings in the underwater environment, encompassing areas such as drag reduction, anti‐biofouling, and corrosion resistance. A critical examination of commercial offerings in the superhydrophobic coating landscape offers a current perspective on available solutions. In conclusion, the review provides valuable insights and forward‐looking recommendations to propel the field of underwater superhydrophobicity toward new dimensions of innovation and practical utility.

## Graphical abstract

Superhydrophobic surfaces hold remarkable potential benefits across multiple industries in contact with seawater. This review focuses on the latest bio‐inspired superhydrophobic surface design and fabrication technologies, breakthrough fabrication strategies for enhanced mechanical, chemical, and, crucially, air plastron durability, and state‐of‐the‐art underwater applications. This review also assesses the commercial superhydrophobic coating products which potentiate future underwater applications.

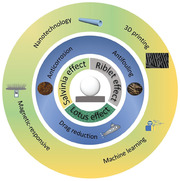

## 1 Introduction

In recent years the emergence of superhydrophobic (SH) materials and coatings has provided ground‐breaking technologies with extensive applications across such diverse sectors as construction, automotive, transportation, aerospace, medical, healthcare, energy, and textiles, amongst others.^[^ ^1^, ^2^, ^3^ ^]^ Although the use of these materials in terrestrial, or in‐air, applications has been the overarching focus, it is in underwater, or submerged, environments where superhydrophobic surfaces (SHS) hold remarkable potential. Their application across multiple industries promises to provide opportunities to improve vessel hydrodynamic efficiency, decrease maintenance demands for equipment and platforms, and enhance the overall performance of undersea infrastructure. For example, underwater SHS can significantly reduce drag for ships and underwater vehicles as they move for the contact between the water and the submerged surfaces is reduced, leading to substantial fuel savings and increased speed and range. Industries such as underwater robotics, offshore energy production, underwater communication cables, subsea pipelines, infrastructure development, and aquaculture can all reap the benefits of underwater SHS through their ability to minimize biofouling and corrosion of their functional subsea structures. The protection of underwater instruments and sensors from biofouling and corrosion by SH materials can enhance the functionality and lifespan of devices during prolonged deployment. These characteristics are particularly valuable for maintaining the efficiency of offshore structures, ensuring uninterrupted communication, safeguarding pipelines, and supporting sustainable aquaculture practices. Additionally, underwater SHS also find utility in diminishing noise,^[^ ^4^ ^]^ facilitating gas collection,^[^ ^5^, ^6^ ^]^ and enabling efficient oil/water separation.^[^ ^7^, ^8^ ^]^ However, unlike in‐air coatings, underwater coatings must contend with dynamic water flow, extreme pressure variations, saltwater exposure, and the constant threat of biofouling.^[^ ^9^, ^10^, ^11^, ^12^, ^13^, ^14^ ^]^ These factors demand innovative and tailored approaches to the design of SH coatings that can withstand the harsh conditions of the marine world.

Most SHS surfaces are prone to damage in real‐life applications owing to their susceptibility to chemical and physical degradation. The surface roughness is generated by the delicate micro/nanostructures, which can be easily damaged by different levels of external stresses, ranging from simple finger‐touches to high‐impact abrasion.^[^ ^15^, ^16^, ^17^ ^]^ When the micro/nanostructures collapse, the trapped air pockets subsequently disappear, increasing the contact area between the liquid droplets and the solid surface and leading to the transition from the water‐repelling into water‐adhesion states. On the other hand, long‐term exposure to the operating environment can cause a series of chemical reactions on SHS, which can alter the surface energy and functional groups. Plastron, a term denoting the layer of entrapped air within the voids of a rough SHS when submerged in water, plays a crucial role in reducing hydrodynamic friction, and resisting biofouling and corrosion.^[^ ^18^, ^19^, ^20^ ^]^ However, sustaining a stable plastron underwater presents a major challenge. Hydrostatic pressure, particularly at greater depths, can compress air pockets and destabilize the plastron, while shear forces from fluid flow, suspended particles, and gas diffusion can further impact plastron stability.^[^ ^21^, ^22^ ^]^

**Figure 1:**
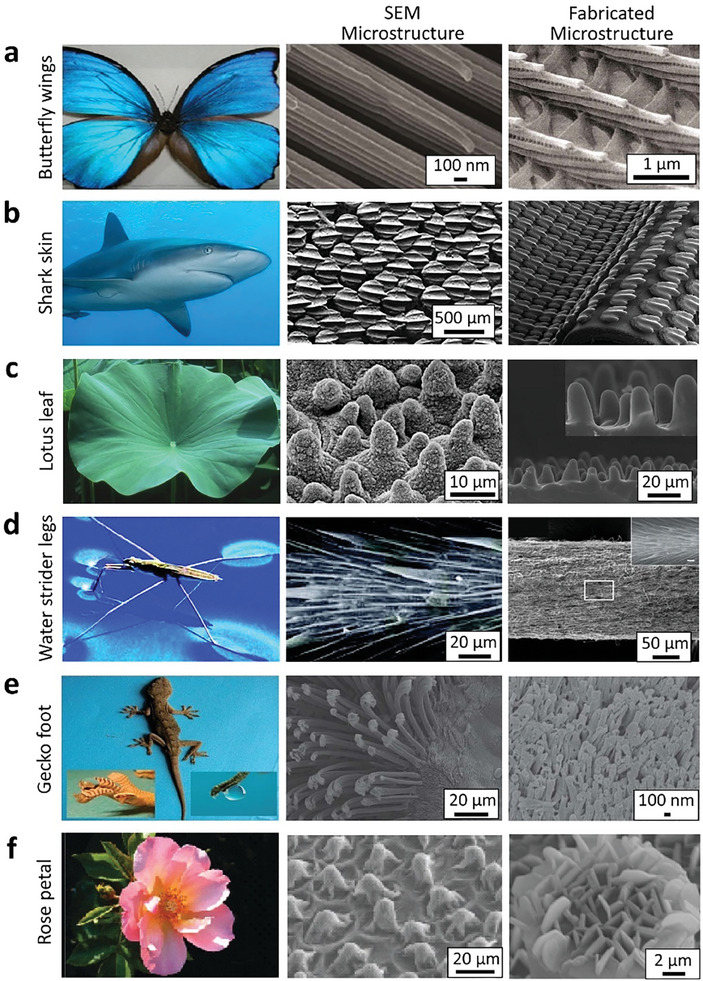
Bio‐inspired SH design in nature, showcasing the commonly adopted natural species, their microstructures and inspired fabricated surfaces like a) butterfly wings. Reproduced with permission.^[^ ^23^ ^]^ Copyright 2010, Elsevier. Reproduced with permission.^[^ ^24^ ^]^ Copyright 2013, Royal Society of Chemistry. b) shark skin. Reproduced with permission.^[^ ^25^ ^]^ Copyright 2018, Wiley. Reproduced with permission.^[^ ^26^ ^]^ Copyright 2017, Elsevier. Digital image of shark was sourced from Wikipedia, under GNU Free Documentation License. c) lotus leaves. Reproduced with permission.^[^ ^29^ ^]^ Copyright 2011, Beilstein‐Institut. Reproduced with permission.^[^ ^30^ ^]^ Copyright 2006, Wiley. d) water strider legs. Reproduced with permission.^[^ ^33^ ^]^ Copyright 2007, American Chemical Society. Reproduced with permission.^[^ ^32^ ^]^ Copyright 2014, American Chemical Society. e) gecko feet. Reproduced with permission.^[^ ^35^ ^]^ Copyright 2012, Royal Society of Chemistry. and f) rose petals. Reproduced with permission.^[^ ^37^ ^]^ Copyright 2010, American Chemical Society. The associated are also highlighted.

**Figure 2:**
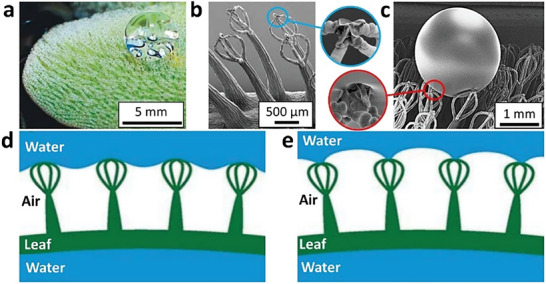
**‘** *Salvinia* effect’, where the eggbeater hair structures with hydrophilic tips demonstrate water‐pinning effects and able to retain air plastron layer for extended duration, simultaneously maintaining its water‐repelling property: a) spherical water drop on leaf surface, b) eggbeater‐shaped structure, c) SEM observation of the interaction between a waterdrop and the leaf surface, d) hydrophobic repulsion and e) hydrophilic pinning. Reproduced with permission.^[^ ^36^ ^]^ Copyright 2010, Wiley.

In pursuit of the development of advanced synthetic SH materials and coatings, researchers have drawn inspiration from nature to enhance the durability and functionality (**Figure** **1**), mirroring the ingenious designs found in the butterfly wings,^[^ ^23^, ^24^ ^]^ shark skin,^[^ ^25^, ^26^ ^]^ lotus leaf,^[^ ^27^, ^28^, ^29^, ^30^ ^]^ water strider leg (*Gerris remigis*),^[^ ^31^, ^32^, ^33^ ^]^ gecko toes,^[^ ^34^, ^35^ ^]^ and more recently *Salvinia molesta*.^[^ ^36^, ^37^ ^]^ These natural models often exhibit hierarchical structures featuring microscale and nanoscale elements that contribute to their exceptional water‐repellent characteristics. For instance, the lotus leaf's surface showcases microscale papillae coated in nanoscale wax crystals, resulting in a rugged and SH texture, known as the ‘lotus effect’.^[^ ^27^ ^]^ Gecko feet rely on densely packed nanoscale hairs and intermolecular forces to securely adhere to various surfaces.^[^ ^34^ ^]^ The ‘riblet’ shark skin roughness,^[^ ^38^, ^39^, ^40^ ^]^ beneficial for underwater applications such as drag reduction and antifouling properties,^[^ ^21^ ^]^ further demonstrates the potential of these bio‐inspired designs. *Salvinia* leaves are equipped with eggbeater‐shaped hair tips that incorporate hydrophilic irregularities and can retain an air layer underwater for up to several weeks or longer, known as the ‘*Salvinia* effect’ (**Figure** **2**).^[^ ^36^ ^]^ Among them, the *Salvinia* design is of pivotal interest to researchers in developing SHS for underwater applications.

In the past decades, biomimetic engineering has been the core design principle for fabricating stronger, more durable and more water‐repelling surface coatings. For instance, Rawlinson et al. pioneered a groundbreaking approach by combining features from seabirds and waterfowl feathers (leveraging hierarchical fibrillar structures for waterproofing through air pocket entrapment) with characteristics of the diving bell spider (which captures air while remaining above water with an aerophilic abdomen).^[^ ^41^ ^]^ This bio‐inspired approach, drawing from various species including birds and spiders, exemplifies the combination of positive traits from each species to design and optimize practical SHS with extended plastron lifetimes. This shift from mono micro/nano patterns to intricate hierarchical nano‐micron scale structures and multifunctionalities reflects the field's ongoing pursuit of remarkable advancements in SH surfaces. There have been several notable reviews in the area of SH materials that have focused on important topics of interest, including the development of coating technologies that are environmentally friendly and sustainable,^[^ ^42^ ^]^ and that use sustainable colloidal materials in their production,^[^ ^43^ ^]^ long term coating robustness and performance,^[^ ^44^ ^]^ mechanical durability,^[^ ^45^, ^46^ ^]^ and progress on submersible SH materials, including the maintenance of the surface plastron.^[^ ^22^, ^38^ ^]^ These reviews collectively highlight the significance of bioinspired concepts, micro/nano structure modifications, and chemical compositions in engineered SH surfaces and their applications. Various techniques, such as lithography, etching, templating, deposition, and more recently, 3D printing, have been employed to realize these design concepts.^[^ ^47^, ^48^, ^49^, ^50^, ^51^, ^52^, ^53^ ^]^ While previous reviews have covered the general development of SH materials, including applications in marine environments,^[^ ^22^, ^38^ ^]^ our review distinguishes itself by focusing on recent advances in designing and fabricating SH surfaces. It explores cutting‐edge strategies to enhance the mechanical, chemical, and, notably, air plastron durability of SH surfaces. Moreover, it delves into the latest applications of underwater SH surfaces, with a specific emphasis on fostering innovation and facilitating practical implementation in marine contexts.

### 1.1 Wetting Models

Understanding wetting models is essential in designing and optimizing SHS due to the complex interplay of surface chemistry, surface morphology, and interfacial forces to achieve high water repellence.

Young's equation (as below) provides a fundamental relationship for the wetting behavior of a liquid on a solid surface.^[^ ^54^ ^]^

(1) $$\cos\theta \;=\;\left(\gamma_{SV}-\;\gamma_{SL}\right)\;\;\;/\;\gamma_{LV}$$

where *θ* is the static contact angle of the ideal solid surface, also known as Young's contact angle or intrinsic contact angle, where *γ_SV_* is the surface tension between the solid and vapor phases, *γ_SL_* is the surface tension between the solid and liquid phases, and *γ_LV_* is the surface tension between the liquid and vapor phases.

This relationship highlights that surfaces with higher surface energy tend to be more wettable (more hydrophilic), while surfaces with lower surface energy tend to be less wettable (more hydrophobic). However, the application of Young's equation follows an assumption that the surface is smooth and homogeneous and that liquid droplet is in thermodynamic equilibrium with the surrounding vapor phase.^[^ ^44^ ^]^ This ideal condition is generally non practical, since most of the surfaces fail to achieve chemical homogeneity and vary in composition, not to mention that high surface roughness is required in the design of SH surfaces.^[^ ^42^, ^55^ ^]^

Alternatively, the Wenzel model (as below) was developed offering insights into how surface roughness impacts wettability.^[^ ^56^, ^57^ ^]^

(2) $$\cos\theta_{w}\;=\;r\cos\theta$$

where *θw* is the contact angle of a droplet on a rough surface, *θ* the contact angle on a flat surface, and *r* the roughness factor defined as the ratio of the actual surface area to the projected surface area.

Based on Wenzel model, the surface wettability is considered to proportionally correlate with surface roughness, which means increasing the roughness of a hydrophobic surface will further enhance its hydrophobicity, and vice versa. Nevertheless, the Wenzel model assumes that the liquid droplet is in full contact with the rough surface, which is not always correct since pockets of air are often observed to be retained on rough surface.

To account for the presence of entrapped air on rough surfaces, Cassie‐Baxter model (as below) was proposed.^[^ ^58^ ^]^

(3) $$\cos\theta_{CB}\;=\;f_{1}\cos\theta +f_{2}\cos\theta_{A}$$

where *θ_CB_* is the water contact angle in the Cassie–Baxter state, *θ_A_* is the contact angle of the water droplet with air, the contact area of the liquid droplet with the rough structures (*f_1_*) and the trapped air bubbles (*f_2_*).

It should be noted that the wetting state of a surface is not always stable. In fact, the Cassie‐Baxter state of a surface can be easily altered and transitioned into Wenzel state when (i) the surface roughness or (ii) surface energy is subjected to changes. This phenomenon is particularly relevant for SH materials, as these surfaces are often exposed to unfavorable conditions.^[^ ^21^, ^22^ ^]^ Therefore, designing SH surfaces with long‐term mechanical and chemical stability is critical to ensure the serviceability of these materials under challenging operating conditions.

## 2 Advancements and Innovations in SH Surfaces Design and Fabrication

The evolution of SH coatings started with the pursuit of the ‘lotus effect’ which involved simply roughening the surface texture of a hydrophobic surface to create micro/nano structures. Advancements in nanotechnology, additive manufacturing, and other technological breakthroughs have allowed researchers to fabricate more complex, well controlled and better performing water‐repelling surfaces. This section will delve into recent novel designs and fabrication methods, such as nanotechnology, magnetic stimulation, additive manufacturing, and machine learning. Through a comprehensive exploration of these techniques, the aim is to uncover their potential and shed light on the strategies for advancing the field of underwater SH coatings.

### 2.1 Pioneering Nanotechnology in SH Coatings

From the previous discussion, it is evident that nano‐micro hierarchical architecture on the SHS is particularly important to ensure water‐repellent and air entrapment properties. Nanotechnology thus plays a crucial role in fabricating SH coatings, capitalizing on the advantageous attributes of nanomaterials such as their high specific surface area, compatibility with polymeric coatings, exceptional mechanical and chemical strength, multifunctionality, and ease of modification. Importantly, this can be achieved cost‐effectively as only a small amount of nanomaterials is required to achieve significant enhancements in material properties.^[^ ^59^ ^]^ Numerous nanomaterials have been applied to fabricate SHS in the past decades, such as graphene oxide,^[^ ^60^ ^]^ zinc oxide,^[^ ^61^ ^]^ titanium oxide,^[^ ^62^ ^]^ boron nitride,^[^ ^63^ ^]^ and silica oxide (SiO_2_),^[^ ^64^ ^]^ amongst others. Selecting appropriate nanofillers and polymers is crucial for fabricating advanced and durable SHS.

**Figure 3:**
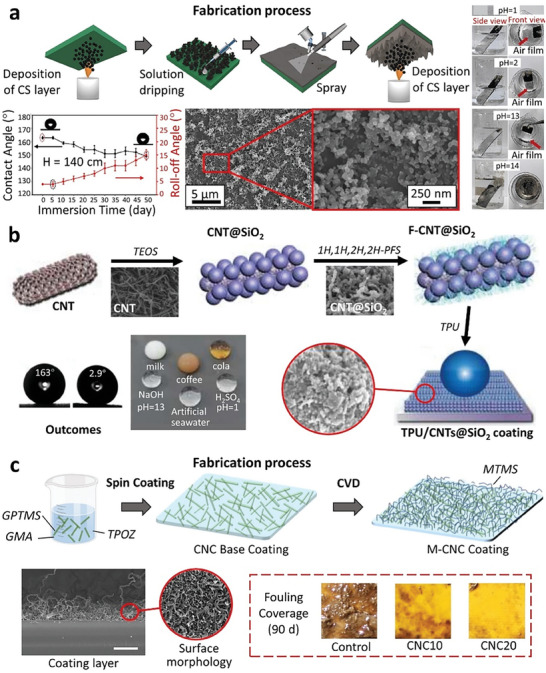
An overview of recent advancements in nanotechnology for designing SHS is presented, encompassing the fabrication process, design concepts, and outcomes: a) The CS composite coating, highlighted for its impressive plastron retention capability and long‐lasting superhydrophobicity at water depth 140 cm for 50 days. Reproduced with permission.^[^ ^68^ ^]^ Copyright 2023, Elsevier. b) CNT@SiO_2_ composite coating illustrating its water‐repelling properties across various liquid media. Reproduced with permission.^[^ ^70^ ^]^ Copyright 2023, Elsevier. c) CNC coating, modified with methyltrimethoxysilane (MTMS), showed exceptional anti‐fouling properties, proven to endure even after 90 days of immersion in a real sea environment. Reproduced with permission.^[^ ^71^ ^]^ Copyright 2023, Elsevier.

Candle soot (CS) nanoparticles stand out as an ideal candidate for surface modification in SH applications due to their excellent water‐repellent properties, cost‐effectiveness and ease of availability (straightforwardly obtained from the incomplete combustion of candles). However, their wide application is hindered by the weak binding between CS and substrate materials, not to mention the carbon nanochains from the CS can be easily removed from the underlying surface by water droplets, which compromises surface stability. Several studies have attempted to overcome these challenges. Qahtan et al. adopted a spray coating method, demonstrating improved water jet resistance and thermal stability compared to direct CS deposition from a candle.^[^ ^65^ ^]^ Xiao et al. introduced methyltrichlorosilane as an adhesive layer between the substrate and SH CS, resulting in high thermal and chemical stability.^[^ ^66^ ^]^ Zhang et al. fabricated a double‐layered polydimethylsiloxane (PDMS) surface with CS deposited on top, showcasing superior superhydrophobicity and promising mechanical‐chemical stability.^[^ ^67^ ^]^ However, underwater testing of CS‐incorporated SHS has largely been lacking, raising concerns about their water‐repellent characteristics after immersion in water. More recently, Wu et al. designed triple‐layer surfaces, including a base adhesive layer, a middle layer and a top SH CS layer, to enhance the CS‐coated surface's underwater mechanical and chemical stability.^[^ ^68^ ^]^ The fabrication process is shown in **Figure** **3**a. The middle layer, made from a mixture of polyester and aprotic solvents, served two important purposes: providing wrinkled deposition sites for the CS and enveloping the CS network to form a tightly bonded structure. Their results demonstrated the prevailing superhydrophobicity after 50 days of immersion at water depth 140 cm, and the existence of a plastron even after 40 days of immersion in solutions with pH levels of 1, 2 and 13, highlighting their good chemical resistance. The effect of CS coating on improving SHS durability (e.g., saltwater, chemicals, and frost attack) was observed in another study.^[^ ^69^ ^]^

The presence of high aspect ratio nanofeatures with good mechanical strength and high elasticity is a prerequisite for achieving the *‘Salvinia effect*’.^[^ ^72^ ^]^ Carbon nanotubes (CNTs) are an excellent candidate due to their outstanding mechanical properties, extremely high aspect ratio (up to 2000), strong adhesion with polymer matrices, and excellent thermal and chemical stability.^[^ ^73^ ^]^ Gu et al. developed a multi‐functional coating by incorporating CNT powders into polyurethane (PU) through a simple and straightforward mixing process.^[^ ^4^ ^]^ The results revealed that the CNT‐incorporated PU coatings exhibited superior corrosion resistance compared with unmodified PU. It is worth noting that addressing dispersion issues is a challenge when using CNTs since they tend to aggregate due to strong van der Waals attraction forces (binding energy ≈500 eV).^[^ ^74^ ^]^ These aggregations hinder their full utilization in composite materials. Hence, effective dispersion strategies should be adopted to ensure the macroscopic performance of CNT based SH coatings. Liu et al. overcame the CNT aggregation through the nanomodifications method (**Figure** 3b).^[^ ^70^ ^]^ They achieved in‐situ growth of SiO_2_ nanoparticles on CNTs using tetraethyl orthosilicate to form CNTs@SiO_2_ nanocomposites which was then modified with silane for enhanced hydrophobicity. The as‐prepared nanocomposites were dispersed in thermoplastic polyurethane (TPU) creating SH TPU/CNTs@SiO_2_ composite coating that exhibited excellent repellence to a variety of liquids, extraordinary corrosion resistance and high durability.^[^ ^45^, ^75^ ^]^ They resisted attack from ions such as H^+^, Na^+^, Cl^−^, SO_4_^2−^ and OH^−^, likely through the trapped air layer existing within the micro/nanostructures of the coating surface.

Cheaper alternatives to CNTs are halloysite nanotubes (HNTs) and cellulose nanocrystals (CNCs). HNTs are natural clay nanoparticles with a nanoscale tube diameter of 15–50 nm and a nano‐micron scale tube length of 300–1500 nm, resulting in an aspect ratio of 6–100.^[^ ^76^ ^]^ As HNTs are hydrophilic by nature and readily dissolved in water, hydrophobic treatment of HNTs is necessary. Feng et al. performed hydrophobic treatment on HNTs through hydrolytic co‐condensation of polysiloxanes (POS) on their surfaces, resulting in POS@HNTs nanocomposites with a core‐shell structure that were spray‐coated onto glass substrates.^[^ ^77^ ^]^ Increasing the silane loading during the hydrophobic treatment increased the surface roughness of the POS@HNTs coating, leading to a higher contact angle (CA) when exposed to droplets from different solutions such as HCl, NaOH, tea, and milk. Additionally, the POS@HNTs coatings exhibited effective oil‐water separation and self‐cleaning functions, which was similar to a study from Song et al.^[^ ^78^ ^]^

Duan et al. combined biomimetic engineering and nanotechnology to fabricate a SH coating using CNCs produced from cellulose, the most abundant biomass in the world (**Figure** 3c).^[^ ^71^ ^]^ Unlike other nanomaterials, CNCs are naturally sourced and have a lower environmental impact. However, they face challenges such as high hydrophilicity and strong aggregation in high‐salt environments which limit their reliability for marine applications.^[^ ^10^ ^]^ In this study, CNCs were treated with a sol‐gel method to create a silylated nanocomposite SH coating that replicated the micro/nanoscale hierarchical ridges and grooves found on mangrove leaves (*S. apetala*). These leaves are known for their strong water repellent and antifouling properties.^[^ ^79^ ^]^ The results clearly demonstrated that the water contact angle (WCA) of the CNC‐incorporated coatings was significantly higher than those without CNCs. 20wt% CNC loading in the coating delivered the best antifouling performance as verified by a 90‐day marine field test (submersed at a depth of 0.5 m).

Despite the promising progress in this area, high aspect ratio nanoparticles are often observed to be randomly distributed and packed in SHS which severely limits their functionality and ability to deliver improved superhydrophobicity and plastron stability. Therefore, more advanced techniques have been explored in order to better control nanoparticle alignment and nano/microstructure patterning.

### 2.2 Exploring The Elegance of Magnetic‐Responsive Design in SH Innovations

Magnetic‐responsive SH materials have gained popularity due to the ability to control patterned surface roughness through magnetic fields, switch between different wettability states, and manipulate the movement of droplets and bubbles, etc. Example applications include SH miniature reactors, magnetic controlled wettability for lab‐on‐a‐chip applications, oil‐water separation, SH yolk–shell nano‐reactors, underwater robots, etc.^[^ ^80^ ^]^ Recently, Li et al. successfully fabricated a magnetic SH coating on paper using a simple and scalable one‐step spray‐coating of a suspension consisting of epoxy resin, PDMS, Fe_3_O_4_, and SiO_2_ nanoparticles.^[^ ^7^ ^]^ The coated paper exhibited excellent water repellence, self‐cleaning, bacteria adhesion resistance, oil water separation, magnetic‐controlled mobility as well as good stability. Hereafter, recent magnetic‐stimulated intelligent SHS designs will be discussed.

**Figure 4:**
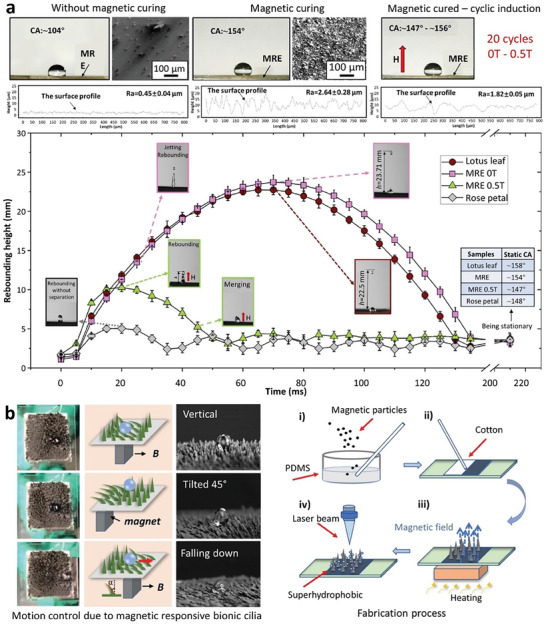
Magnetic‐responsive SH surfaces design and application: a) magnetic curing induced hierarchical surface roughness and cyclic toggling between wetting states (from Wenzel to Cassie‐Baxter and vice versa) by externally manipulating surface roughness through a magnetic field. Reproduced with permission.^[^ ^81^ ^]^ Copyright 2021, American Chemical Society. b) directing the alignment of cilia through the movement of a magnet. Reproduced with permission.^[^ ^84^ ^]^ Copyright 2021, Wiley.

Magnetic‐responsive SHS, inspired by “smart” surfaces with reversible and switchable water‐adhesion properties, hold significant potential for diverse applications involving droplet transmission. Chen et al. drew inspiration from the water‐repellent properties of lotus leaves and the strong adhesion of water on rose petals' surfaces to fabricate magnetic‐responsive SHS with these distinct features.^[^ ^81^ ^]^ This controlled wettability was achieved through the particle‐chain magnetic‐induced deformation on the surface microstructure and surface roughness, resulting in a transformation from an initially rough microstructure with ‘lotus effect’ to a smoother microstructure resembling “rose petal effects” (**Figure** **4**a). In this study, magnetorheological elastomer (MRE) was chosen as the substrate due to its tunable stiffness. When the MRE surface was cured without an external magnetic field, the WCA was ≈104°, and the associated SEM profile showed unevenly distributed surface roughness. Conversely, curing under a magnetic field resulted in a WCA of nearing 154°, showcasing a self‐assembled mountain‐like array surface roughness in the SEM profile. When the magnetically cured MRE surface was exposed to an external magnetic field, the surface roughness and WCA dropped. Importantly, this property proved reversible; cycling the external magnetic field on and off for 20 cycles switched the WCA from ≈156 to ≈147°. Additionally, the trajectories of droplets on four different surfaces – MRE surface with 0 T magnetic field, lotus leaf, MRE surface with 0.5 T magnetic field, rose petal – illustrate the controlled wetting properties of the prepared MRE surface, ranging between the lotus leaf and the rose petal, achieved via the external magnetic field.

On the other hand, Dai et al. produced microcilia morphologies in a carbonyl iron particle/PDMS composite substrate employing a permanent magnet (≈400 mT) and resulting in a WCA of 164° and a sliding angle (SA) of 26°.^[^ ^82^ ^]^ Further, decorating the microcilia array using nanosilica and simultaneous PDMS swelling resulted in an increase in the WCA to 170° and reduction in the SA to 7°. Similarly, Chen et al. employed a magnetically responsive SHS capable of toggling between two distinct surface adhesion phases.^[^ ^83^ ^]^ They used electrostatic air spray deposition and magnetic induction to create a closely packed array of micropillars on a poly(methyl methacrylate) (PMMA) substrate, aligned with the direction of magnetic flux. With the magnetic field, the micropillars stood upright, allowing droplet interaction solely with their tips, resulting in a water adhesion phase with a WCA of 108° and a water‐resistant phase with a WCA of 154°. Without a magnetic field, the loosely packed micropillars allowed droplet penetration, resulting in a transformation from a water‐pinned state (Wenzel states) to a highly water‐repellant state (Cassie states). This system withstood external loads up to 2.2 kPa while maintaining satisfactory performance (WCA = 144° and SA = 12°) under the magnetic field, and quickly transitioned to 110° when the magnetic field was switched off. The collapsed micropillars could be restored by the re‐application of the external magnetic field, demonstrating the surface's cyclical reversal and durability. Fan et al. also developed a magnetic‐responsive SHS that emulated the functional behavior of cilia (**Figure** 4b).^[^ ^84^ ^]^ They achieved this by combining magnetic field assistance and laser etching techniques. The surface demonstrated remarkable capabilities in multidirectional droplet transportation, achieved by controlling the direction of the permanent magnet application and the subsequent manipulation of the cilia. When a magnetic field was applied directly beneath them, the cilia laid flat. Moving the magnet from left to right caused the cilia to sequentially move in the same direction, while simultaneously, the droplet underwent movement as the cilia straightened. The flexible nature of the cilia facilitated bending motions, enabling the transport of droplets in multiple directions. Compared to previous studies focusing on passive modifications of surface characteristics such as morphology and chemical functional groups, the active regulation of wetting properties demonstrated by these “smart” surfaces holds significant value. The real‐time and instant change in wetting and adhesion behaviors offered by these surfaces is highly desirable for applications such as droplet‐based microfluidic chips and microfluidic transportation.

Controlling the motion of bubbles in the underwater environment has been a prominent research area in recent decades due to its importance in industrial applications such as gas collection,^[^ ^85^ ^]^ heat transfer,^[^ ^86^ ^]^ and gas evolution reactions.^[^ ^87^ ^]^ Understanding the directional movement of bubbles is also crucial for designing and optimizing plastron regeneration systems for underwater SHS. Smart manipulation of bubbles can effectively counteract external factors that destabilize the plastron, including buoyancy, hydrostatic pressure, and gas dissolution. Zhang et al. drew inspiration from SH butterfly wings (*Morpho aega*) which enable directional rolling of water droplets and slippery properties of pitcher plants (*Nepenthes alata*) to introduce an anisotropic slippery cilia surface capable of effectively manipulating the motion of bubbles.^[^ ^88^ ^]^ By incorporating fine cobalt powder into the surface, they created a magneto‐controllable system that precisely controlled the multidirectional transport of bubbles using an external magnetic field. This approach addresses the limitations of previous methods, where the controlled motion of underwater bubbles was only achieved unidirectionally through methods such as gradient‐based topography and wettability.^[^ ^5^, ^89^ ^]^

On another front, Wang et al. developed a remarkable SH microrobot capable of performing multiple tasks by responding to different stimuli.^[^ ^90^ ^]^ The microrobot was designed ingeniously to incorporate photo‐responsive graphene and magnetic Fe_3_O_4_ nanoparticles, providing it with versatile and multi‐stimuli‐responsive characteristics, including magnetic field, light, and chemical control. By synergistically employing these control methods, the microrobots effortlessly glided on water, executing precise and controlled trajectory motions such as circular, spiral, and helical movements. Moreover, they demonstrated the microrobots' effectiveness in oil spill recover. Notably, even after undergoing more than six cycles of oil absorption and heating, the microrobots maintained their SH nature (WCA > 150°).

In summary, the untapped potential of magnetic‐responsive SHS offers promising prospects. Expanding their applications beyond the current scope presents an exciting avenue for their advancement and widespread adoption.

### 2.3 Breakthroughs in SH Coatings through Additive Manufacturing

In the past decades, various techniques, including lithography, etching, templating, and deposition, have been employed to create patterned SHS.^[^ ^47^, ^48^, ^49^, ^50^, ^51^, ^52^, ^53^ ^]^ However, these methods have significant drawbacks, such as multiple processing steps (often involving precursors, organic solvents, production of waste materials), and lack of capability of producing complexed geometries. In contrast, high‐precision additive manufacturing technologies such as 3D printing offer a promising solution. By utilizing 3D printing, patterned micro/nano hierarchical geometries (e.g., overlapping, overhang, mushroom like, inward curvature, egg‐beater, re‐entrant, needles, pillars, and hierarchical structures) mimicking complex natural designs (e.g., shark skin, lotus leaves, or *Salvinia molesta* leaf) can be fabricated in a single printing step at the nano‐micron scale. More importantly, the flexibility offered by 3D printing in terms of geometric freedom and dimensions enhances their appeal for design optimization. With the advancement of 3D printing techniques and better accessibility, considerable research effort has been devoted to leveraging various 3D printing technologies for controllable, reproducible, and cost‐effective design and fabrication of SHS.

**Figure 5:**
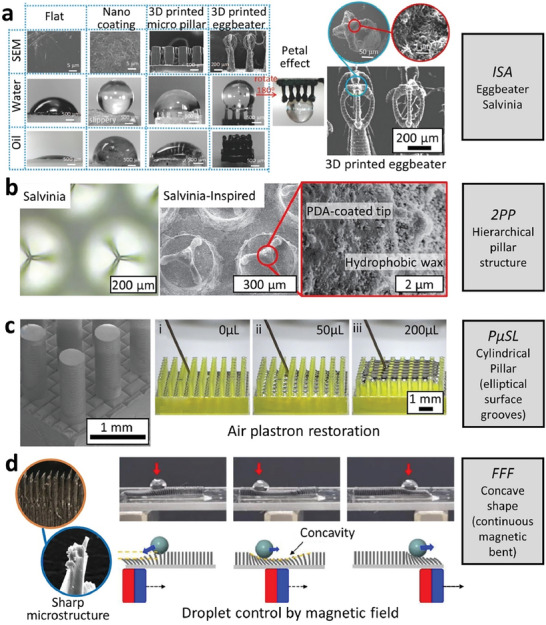
The latest additive manufacturing technologies and their capabilities in replicating/optimizing complex geometries and shapes. a) immersed surface accumulation‐based 3D printing (ISA‐3D) system for replicating the eggbeater shapes of *Salvinia* and a comparison with simple micro pillar design for water repellence. Reproduced with permission.^[^ ^91^ ^]^ Copyright 2018, Wiley. b) a 2PP 3D printing system for producing superhydrophobic hierarchical pillar structures and polydopamine (PDA)‐coated tip. Reproduced with permission.^[^ ^92^ ^]^ Copyright 2022, Nature Portfolio. c) a projection micro‐stereolithography (PµSL) for fabricating cylindrical pillar shapes with convex groove curvature that facilitated plastron regeneration. Reproduced with permission.^[^ ^93^ ^]^ Copyright 2022, American Chemical Society. d) a fused filament fabrication (FFF)‐type 3D printing for creating magnetically controllable micro‐patterned wall arrays for droplet control. Reproduced with permission.^[^ ^94^ ^]^ Copyright 2023, Elsevier.

Inspired by the eggbeater hair tips of *Salvinia* species in micro‐scale, Yang et al. fabricated a SHS using a novel immersed surface accumulation (ISA) based 3D printing process (**Figure** **5**a).^[^ ^91^ ^]^ The printer layered photocurable resin on the substrate following the movement of a light guide tool (resolution of 2.5 µm per pixel). In this study, geometrical features (size, number of eggbeater arms, gap distance) were tailored in a series of designs to investigate their effects on the hydrophobic property and water adhesion on the substrate as well as the mechanical properties. Multi‐walled CNT were added to the resin to remove the accumulated static charges during printing, to enhance the mechanical properties of the eggbeater hairs (the modulus was increased from 161 to 455 MPa with 0.5wt% CNT) and importantly to increase the nano‐roughness of the microstructures thus hydrophobicity. Adjusting the number of eggbeater arms dramatically changed the wettability: WCA < 150°, WCA = 170° and WCA = 152° for 0, 2 and 4 arms, respectively. It was also observed that modifying the number of eggbeater hairs and their gap distance allowed for adjustment of the adhesion forces, which is beneficial for their application in droplet manipulation, oil spill clean‐up, and oil/water separation. Kim et al. 3D printed the original egg‐beater master mold with hierarchical pillar arrays (**Figure** 5b) using Photonic Professional (2PP) GT (Nanoscribe GmbH) by using IP‐S resin on an ITO coated glass, with 1 µm resolution, which highlighted the high meniscus pulling force of *Salvinia*‐like surface, crucial for preserving a stable air‐water interface.^[^ ^92^ ^]^

Inspired by the convexly grooved bases of *Salvinia* leaves supporting eggbeater‐shaped fibrillar outgrowths, Han et al. conducted a comprehensive investigation into the impact of various parameters, such as groove surface curvatures and groove spacings and depths, on the seed gas layer robustness and restoration of their air plastron at the second level grooves.^[^ ^93^ ^]^ The designs were realized using 3D projection micro‐lithograph stereo exposure system (PµSL) and photosensitive resin as the printing material (**Figure** 5c). Through a combination of experimental testing, nanoscale computations, and theoretical analysis, the study revealed the crucial role of groove convexity in designing second level microstructures for stable gas layer and effective underwater air plastron restoration. Elliptical grooves led to a stable and thicker gas layer for all groove depths while V‐shaped grooves, especially shallow grooves, did not support a gas layer. The elliptical design also facilitated efficient gas transport through the interconnected grooves and thus plastron restoration.

More recently, Park et al. conducted an innovative study that combined biomimetic principles, magnetic responsive materials, and 3D printing to develop a magnetically controllable micro‐patterned wall arrays (MCMAs) film (**Figure** 5d).^[^ ^94^ ^]^ They employed a simple and cost‐effective fused filament fabrication (FFF)‐type 3D printing method, which allowed for the rapid and convenient creation of numerous nano‐micron scale cilia structures on the substrate surface. Interestingly, the filament laminating process during FFF‐type 3D printing resulted in a characteristic “staircase effect”,^[^ ^95^ ^]^ which produced a sharp microstructure when the printed structure was cast on PDMS containing magnetic particles. As a result, the surface exhibited extraordinary SH properties without requiring additional post‐chemical hydrophobic treatment. The study demonstrated that MCMA films with optimized geometries can achieve superhydrophobicity (WCA > 150°). By the aid of external magnetic field, a concave shape was formed with continuously sharp bends, allowing efficient droplet manipulation.

Despite these promising advancements, challenges still persist. One major challenge is achieving a dual‐scale roughness at the nano‐micron level, which remains difficult for most commercial 3D printers. As printer resolution increases, costs escalate, printing times are prolonged, and the available selection of materials becomes limited, making the fabrication of large SH materials using high‐resolution printers at this time financially impractical. Another hurdle in 3D printing SHS lies in the fact that the coating degradation through abrasion or underwater attack to the substrate can remove their surface characteristics and degrade their water repellent property. In conclusion, further advancements are necessary to fully capitalize the advantages of 3D printing for SHS, driving industrial commercialization and enabling large‐scale production of such surfaces.

### 2.4 Harnessing the Power of Machine Learning in SH Designs

Despite successful reports on SHS development, design optimization remains a significant challenge. Surface superhydrophobicity depends on various design parameters such as surface roughness, geometry, energy, morphology, and chemical groups. For surface texture design, different morphologies (e.g., pillar, protrusion, stack‐layer) have been adopted, however it remains difficult to determine the optimal topography for the best water‐repellent properties. Even after deciding on the surface topography, details like pillar shape (cylindrical or cubic), width, height, and pitch spacing, which can be modified from nano to micron scale, require further attention. The combination of these parameters leads to different outcomes, adding complexity. For example, experiments with micropillars of varying dimensions and spacing showed that the WCA was significantly influenced by fine‐tuning the dimensions.^[^ ^96^ ^]^ Similar uncertainties arise with other design parameters. Increasing surface energy, for instance, reduces the CA, but how does it affect other performance criteria like adhesive force and durability? The complex interplay of multiple parameters and objectives in the design optimization process leads to complications, necessitating a better understanding of their influence, dominant governing parameters, and potential interactions. However, achieving these objectives using experimental and numerical simulations is resource‐ and time‐intensive.

Machine learning (ML) is a subset of artificial intelligence that involves the use of algorithms and models to analyse, identify, and interpret patterns and rules from datasets. ML enables algorithms to learn and make predictions or decisions without explicit programming and assumptions and presents tremendous value in the design and optimization of SHS.^[^ ^97^ ^]^ ML algorithms can reveal intricate relationships between design parameters and surface properties, facilitating more efficient and precise optimization processes. Furthermore, ML models can continuously learn from past design iterations, enabling ongoing enhancements and the discovery of innovative solutions. This accelerated design process has the potential to reduce the need for extensive experimental efforts while enabling the development of highly effective SHS. It is important to note that the effectiveness of ML models and algorithms relies heavily on the quality and quantity of data used for training. Sufficient high‐quality data is necessary to ensure optimal performance in ML applications. For this reason, most existing studies utilizing the ML tools in the development and optimization of SHS have adopted a hybrid approach, combining ML with experimental numerical methods.

**Figure 6:**
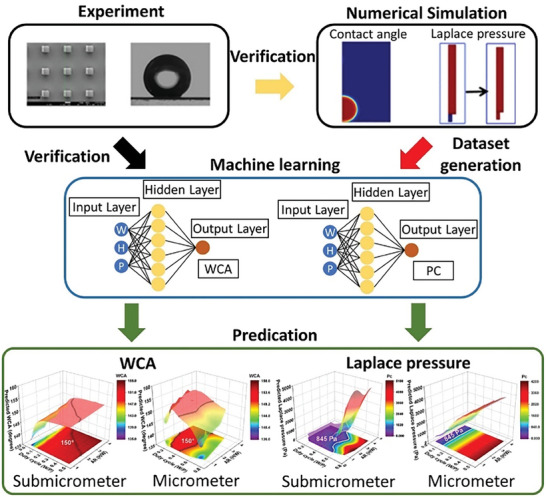
Multistep approaches for designing SHS via combination of experimental methods, numerical simulations, and ML algorithms. Numerical simulations generate dataset for the wetting process and pressure‐induced wetting transition dynamics on different surface topographies using the finite element method (CA and Laplace pressure datasets). The ML uses ANN algorithm for data training. Experiment data is used for verification by cross‐validation between the predicted and experimental results. Depending on the accuracy of cross‐validation, the trained model can be further used for future predictions based on the design space. Reproduced with permission.^[^ ^98^ ^]^ Copyright 2021, American Chemical Society.

Wang et al. developed a design map for finding optimized surface textures for SHS. This map encompasses design parameters that provide optimal choices or trade‐offs to achieve desired objectives.^[^ ^98^ ^]^ The study utilized a hybrid approach, involving experimental methods, numerical simulations, and ML algorithms, as shown in **Figure** **6**. Experimental results validated the FEM and generated a substantial database for ML training (approximately 1000 data points for WCA and around 900 for Laplace pressure). Artificial neural network (ANN) models, inspired by the structure and functioning of biological neural networks in the human brain, were developed and refined using the trained database. The ANN models revealed non‐linear relationships between the topographic design parameters and the WCA/Laplace pressure, with notable distinctions observed between the micrometer and sub‐micrometer length scales. Key findings from the study are as follows: (i) The WCA of sub‐micrometer pillar designs consistently decreases with increasing pitch, while micrometer pillar designs exhibit a nonlinear relationship between the WCA and pitch, (ii) The relationship between the aspect ratio and WCA is not uniform, (iii) The effects of pillar design parameters on the Laplace pressure (a criterion for a stable antiwetting surface) are relatively consistent between sub‐micrometer and micrometer scale pillars but are negligibly affected by the aspect ratio.

Yancheshme et al. studied water droplet interactions with SHS using a hybrid approach of experimental and ML methods.^[^ ^99^ ^]^ This study considered a large number of parameters influencing the droplet behavior, including droplet characteristics (e.g., droplet diameter, density, viscosity, and surface tension), kinetic considerations (e.g., impact velocity), and surface features (e.g., CA, CA hysteresis, and roughness). After analyzing an extensive range of experimental results, ML was employed to predict the droplet behavior upon impact on SHS. The trained model, constructed using the random forest technique, achieved an impressive accuracy rate of up to 98% in predicting droplet behavior. Once again, ML demonstrates its efficacy in material design and prediction capabilities. ML enabled rapid and cost‐effective predictions of droplet dynamics on a wide range of SHS by conducting ≈12,600 tests, which would be time‐consuming and expensive if performed using conventional experimental methods. Zhang et al. significantly advanced our understanding of the relationship between key parameters governing SH performance in nature‐inspired surfaces.^[^ ^100^ ^]^ These parameters include the weight percentage of nanoparticles used in surface coatings and water droplet volume, and multiple objectives including WCA, surface area, and adhesive force. Their study employed an ANN model, providing valuable insights into the design, fabrication, and optimization of multifunctional SH materials. Furthermore, ML has been utilized in other studies to investigate the anti‐freezing characteristics of water droplets on SHS,^[^ ^101^ ^]^ predict the successful integration of the ‘lotus effect’ on such surfaces,^[^ ^102^ ^]^ and design surfaces with enhanced condensation, anti‐icing/frosting, and self‐cleaning properties.^[^ ^103^ ^]^

These works collectively demonstrate the broad applicability of ML in advancing research and development of SHS. Considering the more complicated environmental factors for underwater SHS applications, such as current flow and hydrostatic pressure, ML offers a more resource and time efficient avenue for unlocking new possibilities.

## 3 Insights into Recent Approaches in Obtaining Durable SH Coatings

Despite the intensive effort and substantial advancement in developing SHS, the durability aspect of such surfaces remains elusive in regard to (i) surface roughness/morphology failure and/or (ii) disintegration of the low‐surface energy chemical functionalized coating. Direct mechanical damage, including abrasion, shear, dynamic impact, and adhesion failure, can significantly reduce their durability. Additionally, exposure to chemical stimuli including extreme pH and high salinity may cause chemical degradation of the coating materials and/or change the binding strength between various SHS coating compositions and the substrate. Collectively, these influencing factors can compromise the superhydrophobicity of surfaces by removing the surface morphology or microstructure, revealing layers underneath with non‐hydrophobic chemistry or geometric characteristics.

When it comes to underwater applications, other than mechanical and chemical degradation, the air plastron durability under dynamic conditions including dynamic water flow, hydrostatic pressure, seawater corrosion, and micro‐bacterial attacks plays a significant role in determining the long‐term performance of these coatings. Besides air diffusion and other external stimuli, hydrostatic pressure induced wetting transition is a major issue affecting the plastron lifetime, which occurs quickly when the hydrostatic pressure overcomes the opposing capillary pressure and pressure of the retained air layer in SHS.

While mechanical and chemical durability have been generally assessed for most SHS studies and has been well reviewed,^[^ ^45^ ^]^ plastron stability is not well researched. This section will critically review the latest progress in the latter area in terms of favorable structural and chemical features of SHS, assessment methods and performance.

### 3.1 Strategies in Enhancing Mechanical and Chemical Durability

Enhancing surface topology and chemistry of SHS to withstand structural forces and chemical attack has been a continuous focus of researchers to prolong the retention of geometric features and hydrophobic functional groups.

Volumetric SH coatings provide certain effectiveness to maintain SH characteristics even under the influence of mechanical and chemical degradation.^[^ ^104^ ^]^ This is because following damage the exposed parts of such coatings are similar in texture and functionality to the top/undamaged layer. Liu et al.,^[^ ^70^ ^]^ for example, fabricated a SH TPU/CNTs@SiO_2_ composite that was capable of reproducing micro/nanoscale hierarchical roughness after mechanical abrasion due to the chemical modification of the bulk structure. Guo et al. fabricated a self‐healing SHS by aerosol‐assisted layer‐by‐layer chemical vapor deposition of epoxy resins and PDMS polymer.^[^ ^105^ ^]^ The surface demonstrated excellent durability and maintained superhydrophobicity even after a long period of exposure to different mechanical and chemical attacks. An intriguing feature of this surface was the recovery of its surface roughness, which would otherwise typically be compromised by external damage. The recovery was enabled by the presence of shape memory polymers dispersed throughout the bulk structure, facilitating thermo‐triggered healing mechanisms. The trade‐off of this technique is the significantly higher production cost over conventional coatings.

Multi‐level micro/nanostructures and strong bonding with the substrate convey excellent mechanical and chemical durability and maintenance of superhydrophobicity. For example, WCA of the TPU/CNTs coating dropped to 136° after 130 cycles of sandpaper abrasion (600 grit, 4 kPa) with 75.9 mg weight loss.^[^ ^70^ ^]^ In contrast, the WCA of the TPU/CNTs@SiO_2_ coating remained 157° after 130 cycles with only 25.8 mg weight loss. The friction‐induced shear and tape peeling forces may be dissipated to a large extent due to the multilevel micro/nanostructure, with the composite capable of maintaining the micro‐nanoscale hierarchical roughness after mechanical abrasion due to the chemical modification of the bulk structure. The TPU/CNTs@SiO_2_ coating also showed excellent chemical stability: superhydrophobicity remained (WCA > 150°, roll off angle < 10°) after immersion in H_2_SO_4_ (pH 1), NaOH (pH 13) and 3.5% NaCl for 240 h. Similarly, the nano silica decorated SHS PDMS/SiO_2_ microcilia array significantly outperformed its counterpart without nano‐silica decoration.^[^ ^82^ ^]^ The former was tested to be stable in 9 M HCl, 0.9% NaCl, and NaOH (pH = 14) for 90, 90 and 5.5 h, respectively, and remained SH after 500 cycles of sandpaper abrasion under a pressure of 7.6 kPa, 5 min high speed water jet impact ≈6.5 m s^−1^, 50 h static pressure under 1.5 kg mass, and 1000 cyclic compression via loading and withdrawing of 1 MPa external pressure. The elasticity of the PDMS matrix, the multi‐level micro/nanostructures, along with the robust adhesion of the silica nanoparticles were believed to contribute to the observed outstanding performance.

SHS designs integrating rich micro/nanostructures and elastic micro‐support was proved to dramatically improve the SH coating's reversible deformation to resist mechanical impact. Inspired by the *Calliteara pudibunda* which presents highly elastic micro‐features on it surface, Liu et al. constructed an ultra‐durable SH polyphenylene sulfide‐SiO_2_‐graphite‐fluororubber coating with repairable microstructures.^[^ ^106^ ^]^ The novelty lies in combining abundant thermally expanded graphite micro/nanostructures and elastic fluororubber, the latter serving as elastic micro‐supports that dramatically improve the coating's compression resilience. In this study, hydrophobic expandable graphite@SiO_2_ composite nanoparticles were prepared and then suspended in resin in the presence of elastic fluororubber and spray‐coated on substrate with pre‐coated resin. The coating was then heat treated at 300°C to achieve rapid expansion of graphite. The as‐prepared SHS presented a WCA of 154° and a SA of 3°. The coating remained SH even after 2000 abrasion cycles under a pressure of 125 kPa. Li et al. further verified the enhanced elastic buffering capacity of PDMS as an intermediate layer in SHS, relative to PU and epoxy resin.^[^ ^107^ ^]^ In another study,^[^ ^6^ ^]^ 100 µm PDMS was used as a buffer layer between the substrate and the top SH coating comprising lauric acid, CNTs, TiO_2_ nanoparticles, PDMS and epoxy resin. CNTs here contributed to a dense network structure and provided additional impact resistance to the coating. The superhydrophobicity remained after 350 cycles of 90° bending testing, 1000 cycles of sand abrasion tests, 4H pencil hardness test, and 1500 cycles of sand particles impact test from a 0.5 m height.

Strong adhesion between the SH coating and the substrate is equally important to the mechanical durability of the coating itself. Using epoxy resin as a base coat on substrates, a simple spray‐coating of a suspension of SiO_2_ nanoparticles/PDMS/epoxy resin resulted in a scalable and mechanically durable SH coating.^[^ ^108^ ^]^ The pre‐coated epoxy resin built the interfacial adhesion between the substrate and the SH coating as well as simultaneously strengthening the top coating. In addition to chemical durability, the as‐produced SH coating retained superhydrophobicity after being impacted with faucet water for 1 h, one month of outdoor exposure, and 5 m of sandpaper abrasion under 500 g load.

**Figure 7:**
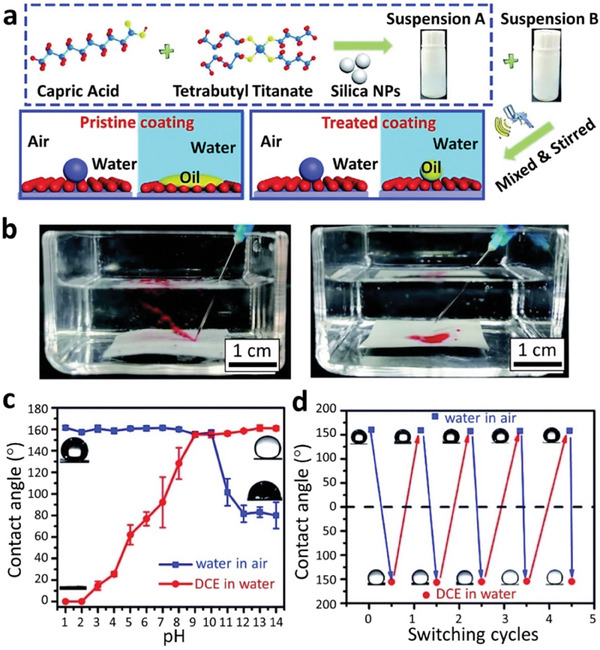
Durable SH coating with simultaneous underwater superoleophobic and in‐air SH properties a) Schematic representation for the preparation process of SH coating with both underwater superoleophobicity and in‐air SH properties. b) Digital images of oil repel from the treated surface and rapidly wet the pristine coating surface. c) CA of water (in‐air) and 1,2‐dichloroethane (underwater) after the coatings were treated in different pH solution media. d) High reversibility of surface in air and underwater displayed by switching cycles of superdewetting states. Reproduced with permission.^[^ ^109^ ^]^ Copyright 2021, The Royal Society of Chemistry.

In some designs, artificial degradation of surface structures yields positive outcomes rather than compromised performance. For example, Sun et al. created a SH coating with simultaneous underwater superoleophobic and in‐air SH properties (**Figure** **7**).^[^ ^109^ ^]^ Upon exposure to controlled corrosion attack (i.e. immersion in weakly alkaline solutions within the pH range of 8–10), the cleavage of titanium‐carboxylate coordination bonding resulted in the formation of hydrophilic defect sites. These defect sites effectively absorbed thin water layers and hindered the spread of oil underwater, leading to a high CA of oil. Nonetheless, the coating maintained its in‐air SH properties since most of the surface still consisted of hydrophobic functional groups capable of counteracting the hydrophilic effects of minor defect sites. The reversible and switchable superdewetting property, observable in both air and water, remained intact even after subjecting the sample surface to aging at 80°C for 2 days, displaying highly reversible behavior with no discernible changes in contact angles. Similarly, Zhao et al. developed a surface by combining hydrophilic aluminum phosphate with SH titanium dioxide nanoparticles, resulting in a heterogeneous chemistry with mainly hydrophilic and hydrophobic regions, thus forming a resilient surface exhibiting superhydrophobicity and underwater superoleophobicity.^[^ ^110^ ^]^

Through a close examination of the latest research in this field, we can acquire valuable insights into the feasibility and applicability of deploying these newly developed surfaces in real‐world applications. Nonetheless, a major challenge in objectively comparing the durability of different SH coatings arises from the lack of standardized testing methods. A comprehensive compilation of testing methods and their noteworthy outcomes concerning the mechanical and chemical durability of SH coatings is provided in Table S1 (Supporting Information). This compilation includes key aspects like adhesion, abrasion resistance, hardness, dynamic impact resistance, thermostability, corrosion resistance, and UV/weathering resistance, covering recent years. Apparently, the wide variation in test conditions and apparatus employed by different research groups restricts the ability to make fair and direct comparisons among the durability of distinct SH coatings. This discrepancy underscores the necessity for harmonizing the testing methods used to assess the durability of SHS. By establishing a unified and standardized approach, researchers will be able to evaluate the long‐term performance of SH coatings more accurately and facilitate meaningful comparisons between different materials and studies.

### 3.2 Noteworthy Research on Improving Plastron Durability

The limited experience and effort in this field can be attributed to the inherent complexities of replicating intricate underwater environments in laboratory‐scale experiments. The constraints imposed by equipment and resource limitations further exacerbate the challenge. Consequently, the forthcoming section aims to shed light on the latest most noteworthy and highest‐performing contributions from the research community on improving plastron durability. Moreover, this section will elucidate the limitations encountered during these assessments.

Babu et al. utilized the similarities of vertically aligned CNTs with the hair‐like structures in *Salvinia* species to fabricate SHS with improved plastron durability.^[^ ^111^ ^]^ Compared to the random growth patterns of CNTs, vertically aligned CNTs were more structurally arranged for capturing and retaining air bubbles as confirmed by confocal microscopy measurements. While as‐prepared CNT coatings only exhibited underwater superhydrophobicity for a brief period, the vertically aligned CNT surfaces demonstrated significantly extended plastron durability, as indicated by a prolonged silvery shine that persisted for several hours. However, the actual underwater conditions in which the surfaces would be used were not taken into account. This could limit the reliability and practicality of applying such surfaces in real‐life scenarios.

Tricinci et. al., presented a novel technique to accurately fabricate the morphology and hydrophilic‐hydrophobic chemical nature of the *Salvinia Molesta*.^[^ ^112^ ^]^ An integrated microfabrication method of direct laser lithography with microcontact printing was adopted in their study, overcoming the limitations of low spatial precision and resolution from conventional microfabrication techniques. For the first time, both the morphology and the chemical nature of *Salvinia* were translated into a synthetic surface. Furthermore, utilizing this powerful fabrication technique, the authors were able to optimize the geometries by preparing hair structures with different filaments and comparing their performance. The optimal design was demonstrated to be SH (WCA up to 170° and tunable adhesion with roll‐off angle from less than 10° to 90°) and proved to possess a remarkable underwater air retention capability, sustained by a stable Cassie‐Baxter state under external hydrostatic pressure up to 4 atm.

Ranjan et al. designed a SHS through the CS coating of a silicon substrate with a porous nanochannel geometry and pretreated with silicon oil.^[^ ^69^ ^]^ Plastron indicated by the silver appearance remained noticeable on the as‐prepared SHS after being submerged under 10 cm of tap water for 30 consecutive days, and the superhydrophobicity remained unchanged from initial values (WCA 159° and roll off angle 3°). Subsequent application of a tap water jet up to 10.3 m s^−1^ incurred no noticeable change in WCA and 360 h exposure in 3.5% NaCl led to a very slight reduction in WCA though an increase in roll off angle to 5°. Additional benefits of the current design are the good organic compatibility and more importantly easy regeneration. Together this presents a promising multifunctional, robust and scalable SHS.

Taking inspiration from the fibrous structure of water strider legs, Tang et al. fabricated a SHS with a micrometer‐scale conical fiber array (M‐CFA).^[^ ^113^ ^]^ The results revealed that the M‐CFA surface was able to capture larger volumes of air bubbles and maintain the trapped air pocket for a longer lifespan (≈41 days) at a liquid depth of 13 cm, compared to the nanometer‐scale conical fiber array (≈10 days) and micrometer‐scale cylindrical glassy fiber array (≈2 days). The presence of a micrometer‐scale asymmetric confined space between fibers created a significant disparity in Laplace pressure, effectively propelling condensed droplets away, while the tips of the fibers assist in pinning the air pocket underwater for an extended duration. Zhang et al. mimicked the hairy structure of cilia through electrostatic flocking and subsequent surface modification of nano‐silica modified PDMS.^[^ ^114^ ^]^ This resulted in a SHS with remarkable water‐repellent properties (WCA = 163°, roll‐off angle = 0°). Notably, the surface retained the plastron phenomenon even after being submerged for a month under normal atmospheric pressure.

**Figure 8:**
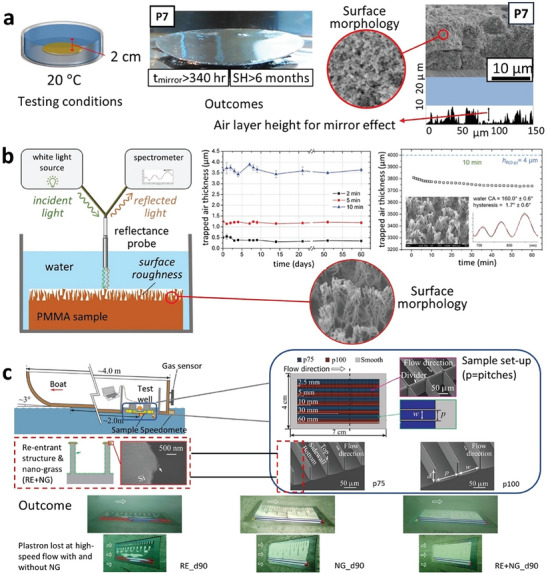
SH designs exhibiting stable air plastron and the testing approaches. a) shallow water (20 cm) testing the plastron stability on P7 (organosilica modified with PMDS through physisorption) coated glass. Reproduced with permission.^[^ ^115^ ^]^ Copyright 2017, American Chemical Society. b) real‐time monitoring and quantification of underwater superhydrophobicity of O_2_ plasma etched poly(methyl methacrylate) (PMMA) film. Reproduced with permission.^[^ ^116^ ^]^ Copyright 2022, Wiley. c) plastron stability on re‐entrant nano‐grass (RE‐NG)‐covered micro‐trench superhydrophobic surfaces tested using the most comprehensive approach that considers a wide range of real‐life environmental factors during testing (13‐foot depth in brackish water at a sea mouth, air saturation level, water flow, shear, turbulence fluctuation, biofouling etc.). Reproduced with permission.^[^ ^20^ ^]^ Copyright 2023, Cambridge University Press.

The SHS with longest demonstrated air plastron durability was achieved by Martinez‐Gomez et al. using PDMS modified nano‐silica particles via physisorption (**Figure** **8**a).^[^ ^115^ ^]^ The existence of an air plastron was determined by visual inspection of silvery‐shine from the coated‐glass samples that were submerged underwater. WCA test after removing the samples from underwater suggested that the surfaces can retain superhydrophobicity after six months of immersion in water. Nevertheless, the in situ silvery‐shine of the glass reflecting the presence of air plastron was not observed. Furthermore, the immersion tests were conducted under a shallow water column of only 2 cm, without considering potential disturbance from environmental conditions. In the work performed by Wu et al., the long‐term superhydrophobicity of the developed SHS was determined by measuring WCA and roll‐off angle every 24 h, but with additional considerations for the effects of hydrostatic pressure on the underwater superhydrophobicity.^[^ ^68^ ^]^ Their results indicated that the designed surface could maintain superhydrophobicity for 46 days, 38 days, and 27 days of immersion at depths of 20 cm (≈1.96 KPa), 80 cm (≈7.84 KPa), and 140 cm (≈13.72 KPa), respectively. However, similar to the previous work, only the WCA was measured, rather than directly observing the presence of the air plastron. This method is not recommended as the samples were taken out from underwater when measuring the WCA, making it challenging to judge the real‐time in‐situ SH performance and the existence of the plastron.

The most appropriate and detailed method to accurately assess the existence of the air plastron is the one adopted by Smyrnakis et al. (**Figure** 8b).^[^ ^116^ ^]^ Previous methods that were used to distinguish air layer properties in submerged systems, such as the indirect CA measurement, direct observation of silvery shine of the submerged solid‐liquid interface,^[^ ^117^ ^]^ laser confocal microscopy,^[^ ^19^, ^118^ ^]^ and attenuated total reflectance‐Fourier transform infrared spectroscopy,^[^ ^119^ ^]^ all suffered from major drawbacks of failing to provide real‐time monitoring of air bubble behavior, thus being unable to directly characterize the air bubble layers and visualize the transition from the Cassie to Wenzel states in an underwater environment. For the first time, real‐time monitoring of the underwater superhydrophobicity and the entrapped air layer thickness were achieved through white light reflectance spectroscopy (WLRS). Taking inspiration from ‘*Salvinia* effect’, a SH amorphous PMMA surface with high air bubble retention capability was prepared through plasma micro‐nanotexturing technology that could fabricate complex hierarchical topographies.^[^ ^116^ ^]^ From the results, all the SHS fabricated with hierarchical roughness were found to maintain a stable air bubble layer for at least 60 days. Throughout the 2‐month period, regardless of variations in plasma etching times, the surfaces consistently remained dry, showcasing enduring underwater superhydrophobicity. The differences observed were in the thickness of trapped air bubbles, influenced by variations in surface peak roughness height and their capacity to capture bubbles. While the method of measuring the air bubble was deemed reliable, it is important to acknowledge that these surfaces were only submerged to a shallow depth of a few centimeters. At such limited depths, crucial external environmental factors such as air diffusion and fluid flow are not fully representative of realistic marine conditions. Consequently, the reliability of their performance in actual marine environments remains questionable, as observed in much of the previous research.

The most robust and comprehensive approach, which yields the most reliable results by considering a wide range of environmental factors, undoubtedly lies in conducting actual field tests. A notable example of such a well‐designed experiment is the study conducted by Yu et al. (**Figure** 8c).^[^ ^20^ ^]^ They examined the durability of air plastrons on SHS by subjecting them to testing beneath a 13‐foot motorboat in brackish water at a sea mouth. This field test took into account various influential factors, including pressure, air saturation level, water flow, shear, turbulence fluctuation, and other real‐life environmental impacts on air plastron durability. The experimental setup involved a specially designed test well that housed a testing unit comprising sample surfaces on the boat hull. Two underwater cameras were installed to provide visual monitoring of the plastrons. The air saturation level was regularly monitored using a total gas sensor, while the boat speeds were varied from 2 to 7.2 m s^−1^ with intervals of ≈ 0.5 m s^−1^. Shear stress at different speeds was measured using a dedicated shear stress sensor. This meticulous approach undertaken by Yu et al. ensures a comprehensive evaluation of the air plastron's durability, incorporating various real‐life factors. The results indicated that samples with nanograss features experienced significantly less air plastron loss at high‐speed flow compared to those without. Wu et al. also conducted a real field test in their work to evaluate the functionality of a three‐layer CS coated surface, where the samples were immersed in a natural lake for a duration of 50 days at a water depth of 20 cm.^[^ ^68^ ^]^ The outcome suggested that the coated surface was able to retain its superhydrophobicity and withstand the turbulent flow and micro‐activities in the lake for more than 35 days.

Despite the increasing efforts to improve the lifetime of plastrons to ensure the functionality of SHS underwater, the reported results present a great challenge for their long‐term application as the failure of plastron layers seems inevitable. In addition, the reliability of these results is still questionable as the testing conditions normally failed to match the actual exposure conditions of the surfaces. Strategies for plastron regeneration, therefore, have been investigated to prolong the service life of SHS.

**Figure 9:**
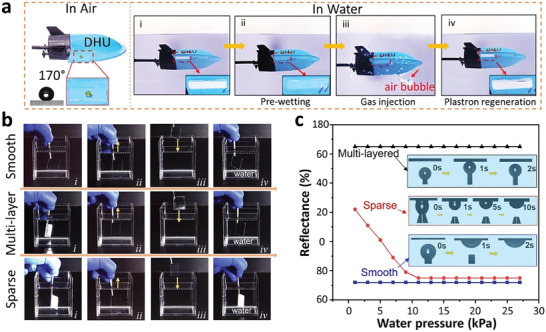
Recoverable underwater superhydrophobicity from a fully wetted state via dynamic air spreading. a) Superhydrophobicity demonstrated by sparse surface structure on toy submarine in air (WCA ≈170°), where air plastron status was indicated by the silvery shine i) immediately upon submersion, ii) during pre‐wetting, iii) during gas injection, and iv) restored plastron after gas injection. b) Changes in plastron when samples subjected to repeated pre‐wetting and regeneration. c) Reflectance of the plastron‐regenerated surface at different water pressures. Reproduced with permission.^[^ ^120^ ^]^ Copyright 2021, Elsevier.

External gas injection has been proven to be the fastest recovery self‐healing system: upon bubble collision with the surface, the injected gas swiftly disperses through the gaps between the surface's rough features. This process enables the restoration of superhydrophobicity within a remarkable timeframe of just 5 s, well before the complete depletion of air pockets.^[^ ^18^ ^]^ One of the most recent efforts include that by Rawlinson et al. who fabricated a SHS with large volumes of voids for air entrapment.^[^ ^41^ ^]^ To extend the survivability of the plastron, a gas bubble stream was injected into the SHSs at time intervals of 2 h via solar‐powered pulsing, which prolonged the lifetime of plastron from 4.8 to 29 days. Nevertheless, it should be noted that the plastron behavior was observed under static water in their work. In another study, Zhao et al. fabricated an aerophilic surface which can act as a respiratory skin that enables the pinning and stable spreading of air bubbles on the skin under submerged conditions,^[^ ^120^ ^]^ suitable for underwater vehicle applications such as submarines. To achieve such an underwater plastron recovery system, the surfaces must be both SH underwater and aerophilic so that the injected air bubble will not escape from the surface due to buoyancy and other external environmental factors. Inspired by the low surface energy sparse hairy structure of the water bug,^[^ ^121^ ^]^ a low‐energy sparse surface structure was prepared by dip‐coating chained silica nanoparticles on a functionalized substrate using amino silane and fluorination treatment. The presence and status of the plastron were assessed using a toy submarine coated with the SH coating (**Figure** **9**). The silvery shine on the boat's surface served as an indicator of the plastron status. Results revealed that gas injection onto the toy submarine's surface successfully regenerated the plastron after pre‐wetting, indicating the potential for these coatings to achieve plastron recovery underwater through straightforward gas injection or collection methods. Furthermore, the recovered plastron resulting from gas injection could withstand >24 h and 120 cycles of ethanol wetting‐bubble injection due to the very low energy barrier (0.4 nN) required for the attachment of air bubbles on the modified sparse surface. However, using optical microscopy, it was clearly observed that the plastron became unstable at high water pressure (25 kPa) and completely disappeared at 40 kPa, attributing to the replacement of the solid‐vapor interface by the solid‐liquid interface as water pressure increased. Overall, even though the gas injection method is reversible and instantaneous in terms of plastron recovery, it heavily relies on a continuous supply of external gas bubbles to the surfaces. It requires integrating an air reservoir with sufficient capacity into the SH coatings, posing a challenge in designing a more complex system.

The development and commercialization of underwater SHS have been met with substantial challenges, including the crucial need to achieve enduring plastron stability, which often involves a trade‐off between the surface CA and long‐term performance. Additionally, replicating realistic underwater conditions for laboratory testing remains a significant hurdle, leading to disparities between lab‐scale success and real‐world conditions. To validate the effectiveness of SHS, comprehensive field testing under realistic operational conditions is imperative, necessitating the establishment of standardized testing protocols. Moreover, the development of multifunctional SHS that do not compromise pre‐existing material functionalities is essential. Addressing these challenges is vital for unlocking the full potential of SH coatings in underwater applications across diverse industries.

## 4 Exploring the Multifaceted Applications of SH Coatings in The Underwater Environment

SH coatings are typically created by combining hydrophobic (water‐repellent) materials with a unique surface structure that creates air pockets, preventing direct contact between the coating and water. Their most promising submerged applications include drag resistance reduction, antifouling and anti‐corrosion. This section will examine latest progress of these applications, delving into their potential to revolutionize various industries and enhance underwater efficiency.

### 4.1 Navigating the Realm of Drag Reduction with SH Coatings

Reducing the environmental impact and enhancing the sustainability of shipping are crucial goals in modern maritime engineering. A significant portion of fuel consumption and emissions can be attributed to a ship's hull hydrodynamic resistance, with friction resistance (drag) alone accounting for ≈60–80% of the total resistance. According to the International Energy Outlook report,^[^ ^122^ ^]^ marine transportation is responsible for at least 12% of global transportation energy consumption. Thus, reducing drag would yield substantial environmental and economic benefits through reducing energy consumption. SHS play a pivotal role in reducing frictional resistance between water and solid surfaces, providing a key solution to this challenge. The air plastron layer created by SHS effectively reduces contact between the surface and water, leading to decreased frictional drag, enabling smoother movement through water. Additionally, the air cushion prevents flow separation, resisting the formation of eddies and vortices that could increase movement drag.^[^ ^38^ ^]^ The reduction in resistance not only enhances navigation speeds for underwater vehicles and surface ships but also results in reduced fuel consumption.

Kim et al. developed a SHS for hydrodynamic drag reduction inspired by the *salvinia* plant's ability to maintain a stable plastron even when submerged for extended periods.^[^ ^92^ ^]^ To replicate the *Salvinia* leaf surfaces, substrates with pillar arrays were prepared employing a soft lithography method followed by capillary‐force‐induced clustering of curved micropillar arrays. The substrate was further coated with wax for hydrophobicity and then with polydopamine to obtain hydrophilic cluster tips. The surface's gently curved structure and localized hydrophilicity through polydopamine coating enable a high meniscus pulling force, maintaining a stable air‐water interface while minimizing water‐solid contact. Through a rotational rheometer test, the researchers measured a substantial effective slip length on the surface, demonstrating an impressive drag reduction of approximately 82%. It should be noted that the *Salvinia*‐inspired surface fabricated in this study demonstrates far superior drag reduction efficiency than some previous studies, normally ranging from 10% to 40%.^[^ ^39^, ^123^ ^]^ Zhang et al. assessed the drag reduction capabilities of their novel cilia mimicking SHS design by rotating the samples in a laminar flow system at shear rates ranging from 40 to 120 s^−1^.^[^ ^114^ ^]^ They measured the torques generated by the surfaces using a plate‐plate rheometer. The results demonstrated a maximum drag reduction efficiency of 28% for the samples.

**Figure 10:**
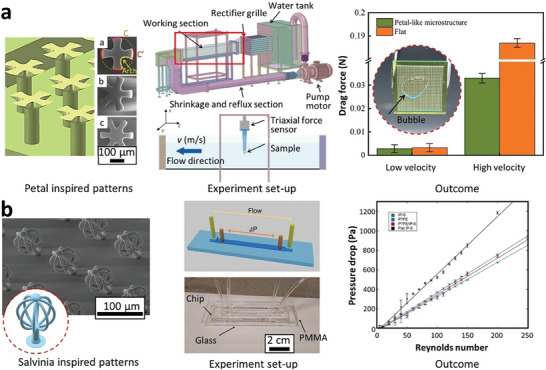
Drag reduction strategies, encompassing experiment setups and outcomes. a) Petal‐like microstructures, fabricated using the PµSL 3D printing approach, demonstrated gas bubble entrapment, resulting in a significant reduction in drag force under high‐velocity flow conditions. The drag reduction was analyzed using a circulating water test bench. Reproduced with permission.^[^ ^8^ ^]^ Copyright 2022, Elsevier. b) *Salvinia*‐inspired SH surface, crafted through 3D laser lithography, exhibited remarkable drag reduction during the test in a microfluidic channel. Reproduced with permission.^[^ ^112^ ^]^ Copyright 2023, Wiley.

Liu et al. measured the drag reduction of the samples using the experimental setup as shown in **Figure** **10**a, where a circulating water test bench was assembled.^[^ ^8^ ^]^ Inspired by the droplet pinning effect of *nepenthes peristome*, a projection micro stereolithography (PµSL) 3D printing system was used to fabricate petal‐like SHS featuring microstructures. A series of microstructures with varied geometrical parameters were prepared to explore parameters of petal number, spacing distance and petal proportion for optimal water repellence, droplet bearing capacity and drag reduction. While a flat design was hydrophilic (WCA = 55°) and micropillar design hydrophobic (WCA = 90°), the petal‐like design was SH (WCA = 160°) and presented superior water droplet adherence owing to the sharp edges and arched curves. The optimal design (4‐petal, 100 µm spacing, and total petal proportion 50%) achieved 58.3% higher droplet bearing capacity than a 0‐petal mushroom design. It was reported that the drag force acting on the petal‐like microstructures surface was reduced by ∼85% under high velocity flow conditions.

Tricinci et al. conducted drag reduction experiments using a molded microfluidic channel as an effective platform for studying boundary flow conditions and detecting microscale interfacial slip (**Figure** 10b).^[^ ^112^ ^]^ A PDMS microchannel with a glass slide patterned with *Salvinia*‐like hairs on the bottom side was employed. Water was pumped through the microfluidic circuit at specific flow rates corresponding to selected Reynolds numbers ranging from 5 to 250. The pressure drop across the channel was measured using a differential sensor. Notably, all experimental acquisitions were conducted in the stationary state within the laminar regime. The results demonstrated that the hydrophobic‐coated *Salvinia*‐inspired surface achieved a remarkable drag reduction factor of ≈41%. The slip length achieved in this study was deemed superior to that observed in previous studies. Even in cases where the slip length of surfaces was higher, they often failed to withstand high hydrostatic pressure, as demonstrated in this study (up to 4 atm).^[^ ^124^ ^]^

Gao et al. innovatively formulated a polyurea/TiO_2_ composite coating that exhibits remarkable SH, self‐healing, and drag reduction properties.^[^ ^125^ ^]^ Through the synergistic self‐healing effects of disulfide and hydrogen bonds within the polyurea, the hydrophobic treated TiO_2_ nanoparticles migrated to the coating surface, contributing to the restoration of superhydrophobicity and ensuring long‐term durability. In an underwater navigation sailing experiment using a submarine model, the composite coating demonstrated approximately 11.28% drag reduction efficiency and exhibited ≈11.04 times higher water loading capacity than its weight compared to an uncoated sample, showcasing its effectiveness in underwater navigation. Su et al. prepared a SH epoxy/polytetrafluoroethylene micro particles coating via simple chemical modification and drop‐coating technique and observed significant drag reduction performance.^[^ ^126^ ^]^

### 4.2 Anti‐Corrosion Capabilities of SH Coatings

Corrosion presents a significant challenge for various marine structures, such as ship hulls and submarines, as their primary construction materials, mainly metals, are susceptible to corrosion. This susceptibility leads to high maintenance costs and environmental concerns. Reported maintenance expenses in the maritime sector account for 20% – 40% of the total operating expenses.^[^ ^127^ ^]^ Corrosion in marine environments is undeniably complex owing to the high salt concentration, wide spectrum of seawater chemistry and presence of biological foulants. Conventional methods like corrosion inhibitors, galvanization, and cathodic protection, including Impressed Current Cathodic Protection (ICCP), each have their limitations, necessitating more effective solutions. ICCP systems, for instance, rely on a power source, exposing them to electrical failures and adding complexity to their application.^[^ ^128^ ^]^ Integrating SH coatings presents an innovative approach to corrosion prevention. These coatings create an air plastron, serving as a protective physical barrier that hinders direct water contact with the substrate, effectively impeding corrosion initiation and progression.^[^ ^129^ ^]^ Notably, the lightweight nature of this thin layer eliminates the necessity for installing external anti‐corrosion devices that would otherwise add weight to the structure, a crucial consideration for large vessels.^[^ ^130^ ^]^

**Figure 11:**
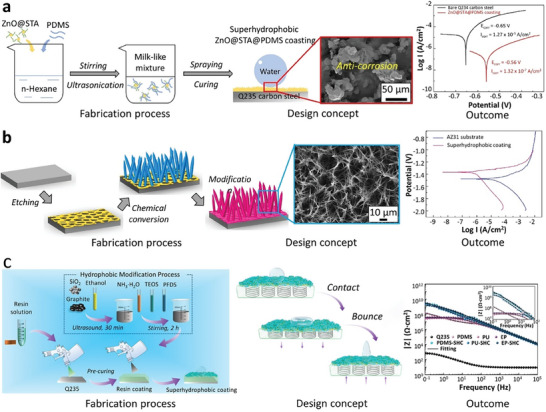
An overview of fabrication processes, design concepts, and their respective effectiveness in combating corrosion through SH technologies. a) Features the ZnO@STA@PDMS SH coating, showcasing a hierarchical nano‐micron roughness with a bump‐porous structure. Reproduced with permission.^[^ ^131^ ^]^ Copyright 2022, Elsevier. b) Demonstrates needle‐like surface nano‐texture achieved through chemical conversion. Reproduced with permission.^[^ ^132^ ^]^ Copyright 2021, Elsevier. c) Highlights the incorporation of polymers with different elastic moduli as an intermediate layer between SHS coatings and substrates. Reproduced with permission.^[^ ^107^ ^]^ Copyright 2023, American Chemical Society.

Zhang et al. prepared a SH ZnO@STA@PDMS coating with strong corrosion resistance using fluorine‐free reagents that are suitable for application in marine submerged conditions (**Figure** **11**a).^[^ ^131^ ^]^ The researchers employed a spray‐coating process to apply a nanocomposite suspension onto a carbon steel substrate, creating a hierarchical nano‐micro scale bump‐porous structure with an extremely low surface energy. This resulted in an optimal WCA of 157° and a SA of 6.8°, signifying the high water‐repellent properties of the surface structure. To evaluate the material's corrosion resistance, electrochemical tests were performed. The surface was immersed in a 3.5wt% NaCl aqueous solution to simulate a marine environment, and electrochemical impedance spectroscopy was utilized to extract essential data, such as corrosion current density (*I*_corr_) and corrosion potential (*E*_corr_), from the potentiodynamic polarization curves. The results from the tests demonstrated that the coated substrate's *I*_corr_ was reduced by two orders of magnitude compared to the bare substrate, indicating an outstanding performance in terms of anticorrosion capabilities.

Using a similar characterization method, Wang et al. investigated the anticorrosion properties of their prepared SH surfaces and made a significant discovery.^[^ ^132^ ^]^ The corrosion inhibition efficiency on magnesium (Mg) alloys, which are highly susceptible to corrosion, reached an impressive 98.14%. To achieve superhydrophobicity on the Mg alloys, they adopted a simple and cost‐effective chemical conversion method, as illustrated in **Figure** 11b, resulting in a WCA of 158.7° and a SA of 1.5°. During their study, the researchers observed a trapped air layer when the surface was immersed in an aqueous solution (as indicated by a shivery shine) due to the needle‐like nanostructure formed via chemical conversion. This observation suggested that the air layer at the interface acts as a barrier, effectively isolating the corrosive medium from the Mg alloy surface. Consequently, this air layer significantly reduced corrosion, highlighting the remarkable effectiveness of the SH coating in protecting Mg alloys against corrosion in challenging environments.

Li et al. engineered mechanically stable and chemically durable SH surfaces by incorporating polymers with varying elastic moduli as an intermediate layer—such as PDMS, PU, and epoxy resin—followed by the application of SH graphite/SiO_2_ coatings (**Figure** 11c).^[^ ^107^ ^]^ Results revealed that all SH coatings exhibited outstanding corrosion resistance, with a corrosion inhibition efficiency of 99.99%. This superior performance was attributed to the robust physical barrier effect of the intermediate layers and the SH characteristics of the surface layers. The study concluded that the presence of intermediate polymer layers, regardless of their elastic moduli, significantly mitigated external impact energy, offering valuable insights for the development of mechanically and chemically durable SH coatings.

Epoxy resin‐SiO_2_‐CNTs‐1H,1H,2H,2H‐perfluorooctyltriethoxysilane coating and curing at room temperature formed SHS on Al plate was shown to exhibit outstanding corrosion resistance.^[^ ^133^ ^]^ The corrosion current density of the coating was 2 and 4 orders of magnitude lower than that of the epoxy resin coating and the bare Al plate, respectively. The bistratal structure consisting of a superficial micro‐nano structure and an underlying porous structure supported air layer stability. The SHS also showed superior mechanical durability: 1400 sandpaper abrasion tests under the weight of 500 g, and mechanical impact with a 1 kg hammer dropped freely from a height of 50 cm. A recent study explored incorporating an anti‐corrosion agent into a 3‐layer SHS coating based on lauric acid intercalated and modified hydrotalcite (La‐LDH) in the form of La‐LDH powder/ PDMS/La‐LDH film.^[^ ^134^ ^]^ A more than 4 orders of magnitude improvement in corrosion resistance was obtained.

Corrosion tests were performed on scratched and unscratched SHS on Q235 carbon steel by Li et al. (consisting of fluoroethylene vinyl ether resin/polycaprolactone as base coat and hydrophobic SiO_2_ as topcoat).^[^ ^135^ ^]^ Each sample was immersed in 3.5 wt.% NaCl solution and characterized using electrochemical techniques. The samples displayed high corrosion inhibition efficiency (≈99.99%) despite the small scratches, while for unscratched samples at least 7 days of effective corrosion protection was presented.

These observations underscore the significance of maintaining a stable air layer in submerged conditions to ensure continued efficacy in corrosion protection. As research in this area continues, SH coatings hold great promise for revolutionizing corrosion protection strategies in the maritime industry and mitigating the significant maintenance costs associated with underwater structures.

### 4.3 Anti‐Fouling Potentials of SH Coatings

Traditionally, the antimicrobial and self‐cleaning properties of SHS have been reported for applications in air, however their use in underwater applications, in particular as biofouling resistant materials and coatings for marine environments, has in recent years been attracting increasing interest. The primary ways of decreasing the attachment and adhesion of microbial organisms and larvae onto SHS are due to the existence of entrapped air layer, low surface energy and a micro/nano hierarchical rough texture.^[^ ^79^, ^136^ ^]^ Ivanovich et al. tested the ability for ship building grade steel (A40S), spray coated with a commercially available SH coating system Rust‐Oleum® Never wet®, to resist biofouling under immersion in the ocean for a period of 7–35 days.^[^ ^137^ ^]^ The coating was deposited via spray coating, resulting in a relatively rough surface (R_a_ of 11.36 µm) with peaks composed of agglomerated SiO_2_ nanoparticles that supported the coating SH characteristics. The authors found the mean WCA to reduce from 151° (0 days) to 121° (21 days), with the coating transitioning to a more hydrophilic state at 28 days (88°–105°) and 35 days (66°–76°). The loss of superhydrophobicity was coupled with an increase in biofouling of the coating, with light fouling present after 14 days easily removed by light fluid flow, while a more strongly adhered light green algal film had grown across the surface of both the SH and control surfaces after 21 days. By 35 days, control surfaces presented significant fouling from bowery crustaceans, sitting polychaetians, bryozoans and sponges, while the SHS exhibited 90% coverage with bryozoans and algae only, indicating a difference in the temporal development and composition of the fouling layer on the SH coating relative to the control.

**Figure 12:**
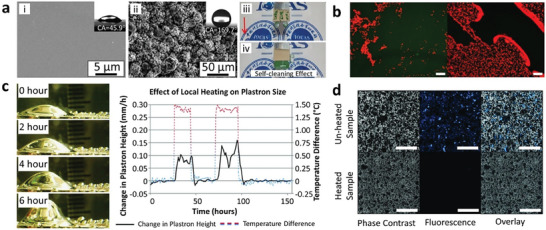
Anti‐fouling through SH technologies a) SEM images of bare Al (i) and hierarchical structured SH surface composed from manganese stearate (ii), with representative water contact angle in insert. (iii–iv) self‐cleaning test, demonstrating the lotus effect that results in the beading of water on the SH surface and the removal of MnO particles. Reproduced with permission.^[^ ^139^ ^]^ Copyright 2016, Elsevier. b) In situ fluorescence microscope images of SH surfaces after 5 hr diatom settlement assay. Left: Image taken without visible bubbles in field of view and, right: a tubular bubble can be seen running diagonally through the field of view. The lens effect can be seen clearly where the fluorescence of diatoms is close to the bubble edge (scale bar: 150 µm). Reproduced with permission.^[^ ^140^ ^]^ Copyright 2013, AIP Publishing. c) Left: Plastron growth on SHS submerged in an open system containing distilled water at 2 hr intervals (scale bar in mm); Right: Average change in height of a plastron measured with heating applied at intervals to demonstrate the temperature dependence of plastron regeneration. Reproduced with permission.^[^ ^141^ ^]^ Copyright 2020, Wiley. d) High resolution fluorescence images of heated and unheated SH samples exposed to a diatom suspension for 5 days. The autofluorescence of the chlorophyll of the diatoms (colored in blue) was used for fouling coverage (scale bar = 500 µm). Reproduced with permission.^[^ ^141^ ^]^ Copyright 2020, Wiley.

Pico‐second laser texturing has been used to produce micron scale surface patterns on stainless steel consisting of either groove (5 µm height, 20 µm spacing) or pit (4.6 µm depth and 18 µm diameter) arrays, with the surfaces then modified with a silicone sol to render them SH (WCA > 150°).^[^ ^11^ ^]^ After 5 weeks incubation in the ocean, both SH surfaces were shown to present a ≈50% lower average microbe attachment area ratio, relative to the unmodified control. Lee et al. fabricated SH petal‐like nanostructure coating on fabrics via a simple dip‐coating process based on the evaporation‐induced self‐assembly of amphiphilic surfactant octadecylamine in the presence of a silica–alumina sol–gel.^[^ ^138^ ^]^ The superhydrophobicity arose from the long alkyl chain of the hydrophobic surfactant and the highly rough hierarchical structure after self‐assembling and curing. The so‐prepared surfaces effectively prevented microbial adhesion during dipping tests. In another study, CF_4_ plasma treated nonwoven polypropylene was significantly less fouled after 7 days immersion in pond water compared to the untreated control material, which correlated to enhanced plastron stability (4.8 ± 1.1 days) relative to the control (2.5 ± 0.9 days).^[^ ^41^ ^]^ In another study, the biofouling resistant properties of a hierarchically structured manganese stearate coating prepared on aluminium via electrodeposition was determined following incubation with the green alga *Chlorella vulgaris* over 3 days under static conditions (**Figure** **12**a).^[^ ^139^ ^]^ The electrodeposited surface was dotted with irregularly spaced voids and pits at the micro‐scale, providing the hierarchical nano/micro roughness characteristic and presented a WCA of 160 ± 3° and surface roughness (R_a_) of 69.3 nm (1×1 µm area). The adhesion of *Chlorella vulgaris* on the control Al surface (≈12.8% surface area) was far greater than the SHS (0.02%), with the authors proposing this to be explained by a low adhesion surface and *lotus* effect (likely during sample removal from the test condition) of the SHS.

The presence of a plastron, or air layer, held to a SHS after immersion in water is an alternate mechanism through which SH surfaces can prevent biofouling organisms interacting with, and adhere to, the surface. Arnott et al. investigated the biofouling performance of their SH silica nanoparticle/polymer coating presenting differing hydrophobic PDMS loading levels that presented variable plastron lifetimes when submerged in water.^[^ ^9^ ^]^ An increase in PDMS loading resulted in reduction in nano‐roughness, WCA and plastron life. Of all samples, the 2.5% PDMS loaded coating exhibited the longest plastron lifetime of ≈65 hrs. The degree of settlement of barnacle (*Amphibalanus reticulates*) and bryozoan (*Bugula neritina*) larvae, and the biofouling diatom *Amphora sp*., all showed an inverse relationship with respect to plastron lifetime, confirming the importance of the plastron for biofouling prevention. The novel SHS developed by Wu et al. employing tri‐layer coating and CS nanoparticles deposition (**Figure** 3a) showed excellent antifouling property.^[^ ^68^ ^]^ The surfaces were tested by submerging them in a turbid natural lake at a depth of 20 cm for several months. While uncoated control surface showed diatom attachment and growth after 8 days immersion and complete biomass coverage after 20 days, the SHS surfaces showed significantly delayed biofouling: biomass attachment 6 days after the edge wetting occurrence on 30^th^ day immersion and full biomass coverage on 46^th^ day.

Another group developed a SH silica nanoparticle/polymer coating that presented varying pore sizes (420 – 765 nm) that readily presented plastrons when incubated in water.^[^ ^140^ ^]^ Laboratory based adhesion experiments using the biofouling diatom *Amphora coffeaeformis* showed the air layer, or air pockets, prevented the cells from approaching and adhering to the surface. The cells were shown to move passively along the air layer/water interface until reaching a wetted area at the edge of the air pocket, at which point the cells were able to contact the surface and adhere. This resulted in the clustering of cells along the wetted boundaries of the air pockets, while no cells were able to adhere to the surface areas protected by the air layer itself (**Figure** 12b). When the surfaces were prewetted using ethanol prior to immersion, the cells were able to easily adhere across the entire surface, highlighting the mode of protection, and a mechanism of potential failure, for SH surfaces presenting plastron layers.

To improve the longevity of the plastron on the surface, and thus the antifouling performance of the SH coating system, a low energy approach to maintain the presence of the plastron on the coating surface indefinitely was developed.^[^ ^141^ ^]^ The thermoregeneration of the plastron was achieved by local heating of the water at the plastron – water interface using a heating element underneath the coating substrate, reducing gas solubility, and thus facilitating the nucleation of gas at the liquid – air interface, and replenishing the plastron (**Figure** 12c). A cell adhesion assay using the fouling diatom *Navicula perminuta* showed that the cell coverage on the unheated glass (48.3%), SH unheated coating (52.6%), and heated glass (47.7%) samples were significantly greater than the SH heated sample (0%), demonstrating the significant potential of this approach (**Figure** 12d).

## 5 Contemporary Commercial Offerings in the SH Coating Landscape

The fabrication of SH coatings has emerged as a captivating research field with vast potential across various applications, including self‐cleaning, anti‐icing, anti‐fogging, anti‐fouling, anti‐corrosion, etc. The increasing adoption of SH coatings in these industries has contributed to the global expansion of the market. Notably, the global SH coatings market has experienced significant growth, starting from $14.2 million USD in 2019 and reaching an impressive value of $19.5 million USD in 2021. Projections indicate further growth, with an expected value of $120 million USD by 2030 and a compound annual growth rate of 25.6% during the forecast period (2022‐2030).^[^ ^142^, ^143^ ^]^ The number of patents related to SHS has experienced a significant surge, witnessing nearly a 3000% growth from 2008 to 2023.^[^ ^144^ ^]^ Despite the immense potential, the transition from laboratory‐scale prototypes to large‐scale commercial applications presents numerous challenges, especially in the field of marine applications. Scalability of fabrication techniques is a primary hurdle, as reproducing early‐stage technologies on a larger scale proves difficult, necessitating solutions for issues such as uniformity, durability, and cost‐effectiveness. Additionally, ensuring the long‐term stability and performance of underwater SH coatings in harsh environments, including UV radiation, saltwater exposure, and mechanical abrasion, poses significant challenges. Furthermore, the compatibility of these coatings with manufacturing processes and various substrates commonly found in marine vessels, such as metals, composites, and coatings, requires attention to ensure seamless integration into practical applications. Regulatory compliance is also critical, as marine coatings must meet environmental and health regulations, requiring the demonstration of safety and compliance for successful commercialization.

Researchers and industry partners have been actively engaged in enhancing and refining the performance of SH coating technologies to overcome associated challenges and drive their commercialization. While SH coatings have gained commercial success in fields like textiles, automotive, construction, utilities, medical, and packaging, their application in the underwater domain appears less popular, offering unexplored possibilities and untapped value. The importance of reviewing the current progress of commercialized or near‐commercialized SH coatings lies in gaining insights into the state‐of‐the‐art technologies and envisaging the future prospects towards transformative potential in submersible applications.

**Figure 13:**
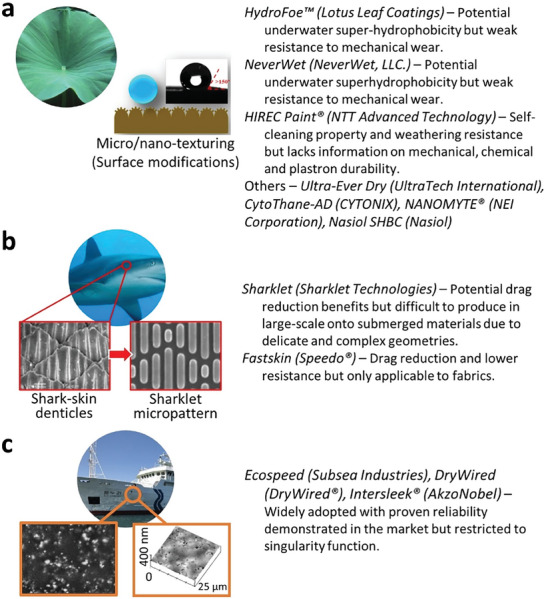
Overview of commercialized SH techniques with multifunctionality. a) ‘lotus effect’. Reproduced with permission.^[^ ^29^ ^]^ Copyright 2011, Beilstein‐Institut. b) ‘riblet effect’. Reproduced with permission.^[^ ^146^ ^]^ Copyright 2018, SouthWest Jiatong University. Digital image of shark was sourced from Wikipedia, under GNU Free Documentation License. and a comparison of c) products based on the conventional design concept with singular functionality. Reproduced with permission.^[^ ^147^ ^]^ Copyright 2019, Elsevier. Reproduced with permission.^[^ ^148^ ^]^ Copyright 2003, American Institute of Physics.

The earliest commercialized SH coatings technology can be traced back to the mid‐2000s with the introduction of products based on the “lotus leaf effect”. Researchers and companies have been keen to replicate the highly textured surface and chemistry of the lotus leaf and develop coatings with similar characteristics. NeverWet, a pioneering company in the field, introduced a SH coating product in 2008 and has progressed rapidly since then on developing a variety of industrial products with improved performance (**Figure** **13**). NeverWet coating products vary based upon the substrate where they are being applied (fabrics, glass, wood, ceramic, metal, concrete, etc) and are applied as one step or two step coatings. The treated surface is dramatically water‐repellent, with water on such surfaces sitting as an almost perfect sphere that will glide off with little friction. The coating on fabrics can sustain multiple cycles of home washes. Ivanovich et al. examined the NeverWet coating for antifouling applications in natural sea water in the Kerch Strait.^[^ ^137^ ^]^ During immersion in seawater at 1 m depth for 3 months, a gradual loss of superhydrophobicity was observed, commencing from the edge of the coating. Surface roughness remained unchanged until the first fouling occurred when the WCA decreased to below 130°. Before reaching that point, the coating effectively reduced friction resistance and prevented the first fouling attachment. Such effectiveness lasted only for 14 days, and it is advised that the main challenge for large‐scale testing of SH coatings in the marine environment lies in their weak resistance to mechanical wear.

There are many other SH products on the market. Ultra‐Ever Dry (UltraTech International Inc.) is a SH wax product that can be applied on a variety of surfaces. The product integrates surface chemistry and texture that completely repels water protecting the underlying substrate from corrosion, contamination and improving durability. The product is highly abrasion resistant (45 Taber abrasion cycles with a loading of 250 g) and presents superior impact resistance (maximum impact speed 22 m s^−1^). However, the coating has poor compatibility to 1 M NaOH (< 2 h exposure), is easily washed off, and the topcoat saturates and wets‐out under submersion. Cytonix LLC, developed an equivalent product – CytoThane‐AD, a two‐part durable super‐hydrophobic coating providing a WCA of 140 to 165° performance in extreme sun and rain. The recommended application of the coat is on marine and ground‐based antenna, radomes etc. The coating is about 80–100 µm in thickness and is expected to maintain its performance for 1–5 years. NANOMYTE® SuperCN Plus (NEI Corporation) is a one component polymeric coating solution providing underlying substrate (plastics, metals, glass, fabrics etc.) superhydrophobicity (fully cured surface WCA > 150°) and improved abrasion resistance. LotusLeaf Coatings developed by HydroFoe™, a SH coating that mimics the surface roughness of lotus leaves using nanotechnology (**Figure** 13). Upon ambient curing, this coating forms an ultrathin layer (< 1 µm) and achieves a WCA of 150– 170°. It could be applied using spin, spray, or dip coating methods, making it easy to apply on various surfaces, including potentially underwater applications. Nasiol SHBC is a two‐layer coating product released by Artekya Ltd. Co in 2013, which can be applied on various surfaces producing a dry film thickness of 8–10 µm (WCA = 171° and SA = 1°).

Extraordinarily durable SH products belong to HIREC Paint® series from NTT Advanced Technology, Japan. The products are sprayable on substrate surfaces after application of an undercoat, producing a 30 µm thick layer with an initial WCA >150°. The coated surface showed <1% reduction in CA after 6 months of outdoor exposure while similar commercially available products showed a 31% reduction. In fact, the excellent water repellence remained even after 3 years’ outdoor exposure (WCA >140°) illustrating the excellent self‐cleaning property. The fabulous self‐cleaning performance is owed to the photocatalytic effect of TiO_2_ nanoparticles in the HIREC 100 coating design. Accelerated weathering test of 2000 h and distilled water immersion test of 100 days at room temperature proved the products’ extraordinary durability – with the WCA remaining above 140°. By far, these products are the best performing and most promising above water SH products available. Nonetheless, information on the mechanical, chemical and plastron durability of the products are not available. Furthermore, the main application of the product has been limited to aboveground facilities for water landing prevention, snow accretion, and ice accretion, while submersible application has not been explored.

All the above commercial products replicate the “lotus leaf effect” – random nano/micro roughness and favorable chemistry owing to easy and versatile application usually by spray application. Such random roughness, however, is not ideal for drag reduction – one of the most desired characteristics for SH coatings for maritime applications such as surface coatings for surface ships, underwater vehicles, and submerged infrastructure. Effective drag reduction relates to the WCA as well as geometrical parameters – roughness and patterning/morphology. Consistent with the “riblet effect” observed on L‐arginine attachment of biofouling. However, the commercialization of riblet patterned SH coatings has been comparatively slower than other bio‐inspired coatings, such as lotus‐leaf coatings. This can be attributed to the more complex and technologically demanding fabrication requirements. The unique microstructure of shark skin, characterized by delicate tooth‐like scales called denticles, poses significant challenges with regards to their accurate reproduction across large areas, as well as the application and deployment of the fabricated surfaces onto submerged materials and structures. These intricate structures demand meticulous attention during fabrication. Fastskin, manufactured by Speedo®, stands out as one of the most widely recognized commercial products that harness the riblet effects inspired by sharkskin in their design concept. This swimsuit has been shown to reduce underwater resistance around the human body by 4%–7%. However, it's worth noting that the fabricated patterns are currently limited to textiles/fabrics and present challenges when attempting to transfer them to other surfaces.^[^ ^145^ ^]^ Sharklet, manufactured by Sharklet Technologies, is a product with microscopic‐sized diamond patterns (≈3 µm tall and 2 µm wide) serving antibiofouling and drag reduction purposes (**Figure** 13). While initially developed for medical applications (e.g., foley urinary catheter and endotracheal tube), it has the potential to be used in various underwater applications. Future efforts should take advantage of advanced techniques to fabricate durable and scalable SH coatings with “riblet effect” for marine use.

It is important to acknowledge that the commercialization of SH coatings for submersible marine use is an ever‐evolving field, and continuous advancements are being made to optimize their performance, durability, and scalability. Ongoing research and development efforts are primarily concentrated on refining the formulation, optimizing application methods, and ensuring the long‐term stability of SH coatings to effectively address the specific challenges and requirements posed by marine environments. In this aspect, some commercialized marine coatings, though not focusing on SH properties, could be adopted to develop durable and multifunctional SH coatings. For instance, Ecospeed, by Subsea Industries, combines glass platelets with resin to form an impermeable multilayer matrix barrier, resulting in excellent antifouling and corrosion resistance capabilities due to the special mix formulation. Other marine coating products such as Drywired® Nanocoating and Intersleek, also possess strong antifouling and anticorrosion properties. By consolidating knowledge in this rapidly evolving field, the future holds the promise of enhanced performance, improved sustainability, and greater efficiency to meet the ever‐increasing demands of the maritime industry.

## 6 Concluding Insights and Forward‐Looking Recommendations

The evolution of SH surfaces reflects an impressive trajectory, evolving from initial inspiration from the “lotus‐effect” to a diverse array of designs that mimic functional biological structures from the water spider, butterfly wings, cilia, and more recently from *Salvinia* species, known for their enduring underwater superhydrophobicity. Notably, fabrication techniques have undergone significant evolution, progressing from the integration of basic nanomaterials into surfaces to the creation of multifunctional, robust nanocomposites. This evolution extends to surface texturing, transitioning from mono micro/nano patterns to intricate hierarchical nano‐micron scale structures. These developments collectively underline the field's continuous pursuit of achieving remarkable advancements in SH surfaces, overcoming traditional boundaries and unlocking new potential applications. This review paper further highlighted the ongoing efforts by the research field in developing better performing SH surfaces with extended service life, including the adoption of the latest technological breakthroughs and concepts, such as machine learning, additive manufacturing, nanotechnology, and stimuli‐responsive mechanisms etc. While the literature covered primarily focuses on SH coatings for above‐air applications, it's imperative not to overlook the inherent potential of adapting and transferring this knowledge to underwater applications. Although the challenges and operating conditions differ, there exist notable similarities in the fundamental principles governing surface wetting and interaction with liquids. The insights gained from above‐air applications can serve as a valuable foundation for developing effective underwater SH solutions. The maritime sector stands to gain substantially from the integration of SH coatings on ship hulls, underwater structures, and offshore installations, leading to improved efficiency, reduced maintenance costs, and enhanced durability. Furthermore, as industries increasingly venture into marine resources and offshore installations, the demand for robust underwater coatings is set to soar. Therefore, even though the field is still in its early stages compared to above‐air applications of SH coatings, it presents a vast array of opportunities waiting to be explored. However, these opportunities come hand in hand with formidable challenges. Other than the long‐term durability concerns and performances of the SH surfaces, the key challenges faced in the fabrication process are identified as follows.

First, developing underwater SH surfaces introduces a more intricate set of considerations. While the principles governing above‐air SH applications emphasize maximizing the WCA and minimizing CA hysteresis for optimal performance, this straightforward relationship becomes less universally applicable underwater, owing to the additional requirement of plastron stability. Unlike in above‐air applications, where maintaining air pockets is relatively straightforward, underwater conditions demand sustained stability of the plastron to ensure ongoing superhydrophobicity. Numerous studies illustrate this complex interplay. For instance, hydrophilic defects intentionally introduced to underwater surfaces create localized water pinning points. As a result, the WCA may decrease, seemingly counterintuitive to conventional above‐air principles. However, this inclusion of hydrophilic defects extends the overall durability of SH performance in submerged environments. This phenomenon, akin to the ‘*Salvinia* effect’, reflects how certain aquatic plants maintain air layers underwater. These hydrophilic defects promote plastron stability by preventing large‐scale plastron detachment, despite the localized reduction in WCA. Such findings underscore the nuanced nature of underwater superhydrophobicity, where trade‐offs between WCA, plastron stability, and long‐term performance must be carefully navigated.

Secondly, identifying and comparing the performance of SH surfaces developed via different methods is difficult, often stemming from the varied and non‐uniform testing methods evident across the literature. This is an issue faced by both above‐air and underwater scenarios. One of the key challenges lies in establishing consistent testing protocols for underwater applications. For example, in underwater applications, different studies utilize diverse testing setups, including submerged platforms, dynamic flow systems, and varying water conditions, resulting in inconsistent and incomparable results. This lack of standardized testing method hampers the ability to draw meaningful comparisons and generalize findings. Additionally, the intricate interplay between surface properties, plastron stability, and underwater flow dynamics adds complexity. The non‐uniformity in testing methods further exacerbates the difficulty of pinpointing the most critical factors affecting underwater superhydrophobicity. To address this challenge, great effort is needed to establish standardized testing methodologies that accurately reflect underwater conditions. By unifying testing approaches, researchers can ensure reliable and comparable results, fostering a clearer understanding of the mechanisms governing underwater superhydrophobicity and accelerating progress in this crucial area of research.

In addition, the replication of realistic underwater conditions in laboratory testing are very challenging due to the complexity of underwater environments. This often leads to a disparity between experimental setups and real‐life scenarios, hampering accurate performance assessment. Moreover, transitioning from lab‐scale success to real‐world applications faces scale‐up issues, resulting in inconsistencies in the effectiveness and durability of SH coatings. Therefore, there is a strong demand in adopting fabrication techniques with both scalability and availability, instead of blindly following the trend of using advanced techniques which gives precise engineered surfaces but expensive or difficult for scaling‐up, or simply using cost‐effective methods that give regular surface features with little control on the repeatability. Hence, there exists a compelling need to embrace fabrication methods that strike a balance between scalability, pattern alignment and availability. Rather than blindly adhering to the adoption of advanced techniques that yield intricately engineered surfaces but are financially demanding or challenging to scale up, or resorting to economical approaches that yield random surface features with limited repeatability control.

Developing multifunctional SH surfaces is also important because it consolidates various benefits into a single layer, reducing the need for multiple coatings with specific functions. This streamlines processes, conserves materials, and minimizes the complexity of application, ultimately leading to resource optimization and cost‐savings. Beyond their water‐repellent properties, these surfaces must not compromise the existing functionalities of the materials they are applied to. Unfortunately, this critical aspect often goes overlooked in research studies. For instance, while the SH properties offer undeniable advantages to sectors like submarines, including enhanced drag reduction, fuel efficiency, and resistance to corrosion and fouling for prolonged service life, there's a notable gap in understanding how SH coatings impact submarine stealth and noise absorption properties. These attributes are pivotal for the submarines' survivability and operational effectiveness. It's imperative that research efforts delve into these multifaceted considerations to ensure that the integration of SH surfaces doesn't hinder other crucial functionalities, leading to an optimal balance of benefits across multiple aspects of application.

## Conflict of interest

The authors declare no conflict of interest.

## Supporting information

Supporting Information
