# Supplementary material for: Recent Advances in Superhydrophobic Materials Development for Maritime Applications
Source: Adv Sci (Weinh). 2024 Feb 25;11(16):2308152. doi: 10.1002/advs.202308152 (PMC11040384; doi:10.1002/advs.202308152)
Supplement: Supplementary file 1 — Supporting Information [file ADVS-11-2308152-s001.pdf]

ADVANCED SCIENCE
Open Access

Supporting Information

for *Adv. Sci.*, DOI 10.1002/advs.202308152

Recent Advances in Superhydrophobic Materials Development for Maritime Applications

Zhao Qing Tang, Tongfei Tian, Paul J. Molino, Alex Skvortsov, Dong Ruan, Jie Ding and Yali Li**

Recent advances in superhydrophobic materials development for maritime applications

Zhao Qing Tang, Tongfei Tian, Paul J. Molino, Alex Skvortsov, Dong Ruan, Jie Ding, Yali Li**

Z. Q. Tang, Y. Li
Centre for Smart Infrastructure and Digital Construction, School of Engineering
Swinburne University of Technology
Hawthorn, Victoria 3122, Australia
E-mail: yalili@swin.edu.au

T. Tian

School of Science, Technology and Engineering

University of the Sunshine Coast

Sippy Downs, QLD, 4556 Australia

P. J. Molino, A. Skvortsov, J. Ding

Platforms Division

Defence Science and Technology

506 Lorimer Street, Fishermans Bend, VIC, 3207 Australia

E-mail: jie.ding@defence.gov.au

D. Ruan

Department of Mechanical and Product Design Engineering

Swinburne University of Technology

Hawthorn, Melbourne, 3122 Victoria, Australia

Supporting information

Supplementary Tables

Table S1 Compilation of testing methods and results for assessing the mechanical and chemical durability performance of superhydrophobic coatings, covering aspects such as adhesion, abrasion resistance, hardness, dynamic impact resistance, thermostability, corrosion resistance, and UV/weathering resistance.

Durability aspects	Method	Outcome	Ref
Adhesion	Tape-peeling test (using 3M, VHB	Withstanding 600 cycles of	[1]

	4910 tape)	adhesive-peeling	
	Tape-peeling test (using 3 M, Scotch 600 tape), applying certain pressure (e.g., 2.3, 16.6, or 90.5 kPa) on the tape and then peeled off after 5 s.	Withstanding 200 cycles of adhesive-peeling at 90.5 kPa	[2]
	Tape-peeling test (using 3M, VHB tape), where tape was rolled over by a 4 kg steel roller twice and then peeled off after 90 s.	Withstanding 30 cycles of adhesive-peeling	[3]
	Tape-peeling test where tape was rolled with a weight of 100 g and then peeled off quickly	Withstanding 300 cycles of adhesive-peeling	[4]
	Tape-peeling tests following ASTM D 3359-09 (using Scotch 810 tape), where a pressure of 24.5 kPa was applied	Withstanding 80 cycles of adhesive-peeling	[5]
	Tape-peeling tests (using Scotch tape), where tape was rolled over with weight of 100 g, and then peeled off quickly	Withstanding 200 cycles of adhesive-peeling	[6]
	Ultrasonic treatment (300 W), where substrate was immersed in ethanol.	Withstanding 30 min ultrasonic treatment	[7]
	Ultrasonic treatment (KH3200E, 150 W, 40 kHz), where substrate was immersed in 100 mL of absolute ethanol.	Withstanding 60 min of ultrasonic treatment	[4]
	Ultrasonic treatment (720 W)	Withstanding 100 min of ultrasonic treatment	[8]
Abrasion	Sandpaper abrasion test with a pressure of ~5 kPa	Withstanding 500 cycles of abrasion on 80 grit sandpaper with abrasion distance of 20 m. 380 cycles of abrasion on 360 grit	[1]

		sandpapers, 420 cycles of abrasion for 600 grit sandpaper.	
	Sandpaper abrasion test (1200 grid) at a velocity of 2 cm/s along the sandpaper under the pressure of 100 g load.	Withstanding at least 30 cycles of abrasion	[8]
	Sandpaper abrasion test (2000 meshes) under a certain pressure (e.g., 2.3, 4.5, or 9.8 kPa)	Withstanding 200 cycles of abrasion on sandpaper at 9.8 kPa	[2]
	Taber abrasion test following ASTM D4060	Withstanding 100 cycles of Taber-abrasion under a 200 g loading wheel	
	Sand abrasion test where the diameter of the sand used is about ⩽ 1 mm	Withstanding 250 times of sand-abrasion	[4]
	Sandpaper abrasion test (800 grit) under pressure of 2.6 kPa	Withstanding 100 cycles of abrasion on sandpaper	[4]
	Linear abrasion test where the coated glass substrate was glued under a load of 100 g moved against a aluminum foil at velocity of 2 cm/s.	CA measured are > 150° up until 100 cm of the linear abrasion test	[9]
	Sand impact test where the diameters of sands used range from 100 to 200 μm, continuously dropped from a height of 50 cm for 5 min (impact energy = 5×10^{-8} to 40×10^{-8} J per grain)	Withstanding sand impact for 5 min	[10]
Hardness	1.5 kg mass was placed onto the surface to give long-term static applied pressure. Cyclic high pressure via loading and withdrawing of 1 MPa external pressure was also performed by a	Withstanding the static pressure after 50 h of loading and 1000 cyclic compression	[7]

	motorized platform		
	Compression test where the materials were compressed at 2 mm/min using a model CMT6103, MTS equipment according to GB/T 1041-92 standard.	PDMS-based coatings can undergo the greatest elastic deformation, followed by PU and EP as the worst.	[11]
	Finger-touching and knife-scratching	Withstanding 140 times of finger friction and 170 cycles of knife scratching.	[12]
	Pencil hardness test where the scratch distance was 6 mm, and the tip load applied was 7.5 N.	Withstanding the scratch test by up to 4H pencil grade.	[13]
	Pencil hardness test following standards GB/T6739-1996	Withstanding the scratch test by up to 6H pencil grade.	[14]
	Hardness tester (KELITI000ZB) was applied, where the force used was 2 N, and hold for 15 s	Surface hardness was improved by laser ablated micro-grooves on the super-hydrophobic coatings.	[15]
Dynamic impact	Water impact test where the water spraying, free-falling water droplet and continuous water stream were applied to substrate	Withstanding 250,000 water droplet impacts (impact speed of 2.8 m/s and an impact pressure of ~3.9 kPa), 100 cycles of water spray impact (impact speed of 3.0 m/s and an impact pressure of ~4.5 kPa) and 600 s of water stream impact (speed of 7.4 m/s and impact pressure of ~27.4 kPa).	[9]
	Water impact tests including free-falling water droplet and water jet applied by high-pressure water gun	Withstanding a water droplet impact at a velocity of ~4.47 m/s, free falling from 100 cm height.<br>Retaining superhydrophobicity after 310 s exposure to water jet at	[16]

		velocity of 8.6 m/s.	
	Water impact test where water flow falling from 45 cm height on the surface inclined at 30° at speed of ~1 mL/s)	Retaining superhydrophobicity after 20 L of water flow impacted on the surface.	[17]
	High speed water jet test to mimic rain impact via streaming tap water on substrate at velocity of ≈ 6.5 1/ms	Withstanding high-speed water jet for 5 min	[7]
	Solid impact test where sand particles were dropped onto surfaces from 0.5 m height.	The CA and SA negligible affected by sand impact even after 1500 cycles	[13]
	Water jet test with a stream of tap water impacting the surface at velocity of ~1 m/s (~0.5 kPa).	Withstanding water jet for at least 30 min	[18]
	Solid impact test where sand particles (size: 200–350 μm) were dropped onto surfaces from 0.30 m height at 50 g/min.	Withstanding the sand impact for 160 min.	[12]
	Rain-simulated water dripping test where deionized water dropping from 30 cm height (1 L/min) on the surface inclined at 0° and 30°, respectively.	Withstanding the water dripping for 240 min (inclination of 0°) and 310 min (inclination of 30°).	[12]
Thermostability	Heat test where 25 - 250 °C heat treatment on the materials for 0 - 24 h	Retaining superhydrophobicity after heated up to 250 °C for 2 h, and after heating at 150 °C for 24 h	[5]
	Hot water test where materials were subjected to hot water ranging from 25 to 95 °.	Withstanding water temperatures up to 95 °, silver mirror effect representing trapped air layer could be observed when the film was immersed in 90 °C hot water.	[7]

	Heat treatment of surfaces ranging from 50 to 350 °C.	Thermally stable up to 350 °C.	[19]
	Heat treatment for 2 h on the surface via drying oven, ranging from 50 to 150 °C.	Thermally stable up to 150 °C.	[15]
	Heat treatment for 2 h via heating plate, ranging from 20 - 300 °C.	Thermally stable up to 300 °C.	[20]
	Hot water test where water heated to 20 - 80 °C was immediately dropped onto surface inclining at 15° for contact angle measurements.	Water repellent even the water droplet was 80 °C.	[20]
Corrosion resistance	pH test where the film was immersed in HCl solution (pH = 1, 9 mol/L), NaCl solution, (pH = 7, 9 g/L) and NaOH solution (pH = 14).	Retaining superhydrophobicity after exposure to HCl, NaCl and NaOH for 90, 90 and 5.5 h, respectively.	[7]
	pH test where the surface was immersed in 1 M H_2SO_4 and 1 M NaOH.	Retaining superhydrophobicity after exposure to H_2SO_4 and NaOH for 12 and 2 h, respectively.	[11]
	Corrosion test where scratched and unscratched samples were immersed in 3.5 wt.% NaCl solution to conduct electrochemical experiments	Displaying high corrosion inhibition efficiency (~99.99%) despite the small scratches. For unscratched samples, demonstrated at least 7 days effective corrosion protection of the coating	[6]
	Corrosion test where samples immersed in 3.5 wt % NaCl solution to measure potentiodynamic polarization of coatings from an electrochemical workstation	The corrosion resistance of coated samples were significantly higher than the bare Al plate, where the corrosion rate and protection efficiency of the coating were 0.274 mm/year and 93.584%.	[19]
	pH test where coated glass slides	Retaining superhydrophobicity for	[16]

	were immersed in a 5 M sulfuric acid (H_2SO_4) solution.	at least 72 h	
	pH test where the coated substrate was soaked in different pH aqueous solutions (2, 7, 12), organic solvents and artificial seawater (2 moL/L NaCl).	Retaining superhydrophobicity for at least 72 h in all immersion solutions.	[8]
UV/weathering resistance	UV irradiation test where an UV source (power: 68 W, wavelength: 253.5 nm) was applied and the distance between the lamp and coating was ~20 cm.	Withstanding continuous 48 h of strong UV irradiation.	[6]
	UV irradiation test where a light source (power = 300 W, λ = 360 nm) was applied and the distance between the light and the samples was 15 cm.	Withstanding continuous 180 h of strong UV irradiation.	[21]
	Actual weathering test where sample was placed at an actual outdoor environment (rooftop), exposing to rain, wind, UV and other outdoor conditions.	Functioning properly even after one month of outdoor exposure.	[21]
	UV irradiation test where a light source with intensity of 7.5 W/m^2 is used	CA measured > 160° and SA < 10° even after 648 h of radiation.	[16]

References

[1] B. Wu, J. Lyu, C. Peng, D. Jiang, J. Yang, J. Yang, S. Xing, and L. Sheng, *J. Chem. Eng.*, **2020**. *387*, 124066.
[2] Y. Li, B. Li, X. Zhao, N. Tian, and J. Zhang, *ACS Appl. Mater. Interfaces*, **2018**. *10*, 39391.
[3] C. Peng, Z. Chen, and M. K. Tiwari, *Nat. Mater.*, **2018**. *17*, 355.
[4] C. Li, P. Wang, and D. Zhang, *Colloids Surf. A Physicochem. Eng. Asp.*, **2021**. *624*, 126835.
[5] P. Wang, Y. Yang, H. Wang, and H. Wang, *Surf. Coat. Technol.*, **2019**. *362*, 90.
[6] C. Li, P. Wang, and D. Zhang, *J. Ind. Eng. Chem.*, **2022**. *110*, 529.
[7] Z. Dai, G. Chen, S. Ding, J. Lin, S. Li, Y. Xu, and B. Zhou, *Adv. Funct. Mater.*, **2021**. *31*, 2008574.
[8] H. Li, Y. Luo, F. Yu, and L. Peng, *Colloids Surf. A Physicochem. Eng. Asp.*, **2022**. *640*, 128449.

[9] I. Torun, M. Ruzi, F. Er, and M. S. Onses, *Prog. Org. Coat.*, **2019**. *136*, 105279.
[10] K. Xu, S. Ren, J. Song, J. Liu, Z. Liu, J. Sun, and S. Ling, *J. Chem. Eng.*, **2021**. *403*, 126348.
[11] C. Li, P. Wang, and D. Zhang, *ACS Appl. Mater. Interfaces*, **2023**, 23875.
[12] Y. Zhao, T. Hao, W. Wu, Y. Meng, X. Cao, Q. Zhang, W. She, J. You, D. Shi, and T. Jiang, *Appl. Surf. Sci.*, **2022**. *587*, 152446.
[13] X. Zang, X. Cao, W. Zheng, T. Zhu, Y. Lei, J. Huang, Z. Chen, L. Teng, J. Bian, and Y. Lai, *J. Chem. Eng.*, **2023**. *451*, 138573.
[14] P. Wang, X. Yan, J. Zeng, C. Luo, and C. Wang, *Appl. Surf. Sci.*, **2022**. *602*, 154408.
[15] H. Wang, L. Tian, J. Zheng, D. Yang, and Z. Zhang, *Tribol. Int.*, **2022**. *173*, 107657.
[16] J. Lyu, B. Wu, N. Wu, C. Peng, J. Yang, Y. Meng, and S. Xing, *J. Chem. Eng.*, **2021**. *404*, 126456.
[17] Z. Liang, M. Geng, B. Dong, L. Zhao, and S. Wang, *Surf. Eng.*, **2020**. *36*, 643.
[18] M. Li, W. Luo, H. Sun, M. Zhang, K. W. Ng, F. Wang, and X. Cheng, *Surf. Coat. Technol.*, **2022**. *438*, 128367.
[19] D. Lin, X. Zhang, S. Yuan, Y. Li, F. Xu, X. Wang, C. Li, and H. Wang, *ACS Appl. Mater. Interfaces*, **2020**. *12*, 48216.
[20] J. Wang, Y. Zhang, and Q. He, *Sep. Purif. Technol.*, **2023**. *306*, 122423.
[21] X.-J. Guo, D. Zhang, C.-H. Xue, B.-Y. Liu, M.-C. Huang, H.-D. Wang, X. Wang, F.-Q. Deng, Y.-P. Pu, and Q.-F. An, *ACS Appl. Mater. Interfaces*, **2023**. *15*, 4612.
